# Efficient CRISPR/Cas9-Mediated Gene Knockin in Mouse Hematopoietic Stem and Progenitor Cells

**DOI:** 10.1016/j.celrep.2019.08.065

**Published:** 2019-09-24

**Authors:** Ngoc Tung Tran, Thomas Sommermann, Robin Graf, Janine Trombke, Jenniffer Pempe, Kerstin Petsch, Ralf Kühn, Klaus Rajewsky, Van Trung Chu

**Affiliations:** 1 Max-Delbrück-Center for Molecular Medicine, Berlin, Germany; 2 Berlin Institute of Health, Berlin, Germany

**Keywords:** CRISPR/Cas9, ribonucleoprotein, RNP, adeno-associated virus, AAV, non-homologous end joining, NHEJ, homologous recombination, HR, hematopoietic stem and progenitor cells, HSPCs, high efficiency, gene knockin, gene repair

## Abstract

Mutations accumulating in hematopoietic stem and progenitor cells (HSPCs) during development can cause severe hematological disorders. Modeling these mutations in mice is essential for understanding their functional consequences. Here, we describe an efficient CRISPR/Cas9-based system to knock in and repair genes in mouse HSPCs. CRISPR/Cas9 ribonucleoproteins, in combination with recombinant adeno-associated virus (rAAV)-DJ donor templates, led to gene knockin efficiencies of up to 30% in the *Lmnb1* and *Actb* loci of mouse HSPCs *in vitro*. The targeted HSPCs engraft and reconstitute all immune cell lineages in the recipient mice. Using this approach, we corrected a neomycin-disrupted *Rag2* gene. The Rag2-corrected HSPCs restore B and T cell development *in vivo*, confirming the functionality of the approach. Our method provides an efficient strategy to study gene function in the hematopoietic system and model hematological disorders *in vivo*, without the need for germline mutagenesis.

## Highlights

• CRISPR/Cas9 RNPs and AAV-DJ donor vectors lead to efficient HR in mouse HSPCs
• No off-target activity was detected when sgRNAs with high specificity were used
• Gene-targeted HSPCs fully engrafted and repopulated all immune cell lineages *in vivo*
• Rag2-repaired HSPCs restore B and T cell development *in vivo*

## Graphical Abstract

**Figure:**
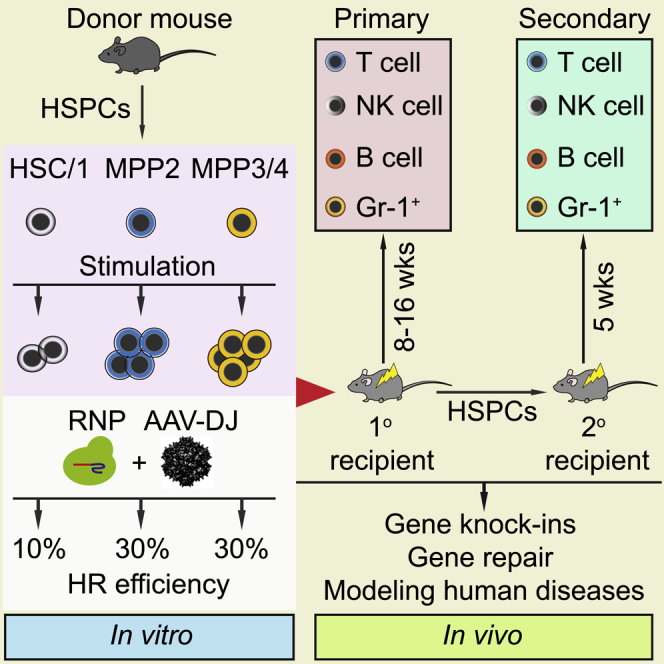

## Teaser

Using CRISPR/Cas9 and AAV-DJ vectors for donor template delivery, Tran et al. achieve high homologous recombination efficiencies (median of 30%) in mouse hematopoietic stem and progenitor cells (HSPCs). The targeted HSPCs engraft and repopulate all immune cell lineages after primary and secondary reconstitution of immunodeficient recipients.

## Introduction

Hematopoietic stem and progenitor cells (HSPCs) are self-renewing and multipotent cells that repopulate the entire hematopoietic system upon transplantation into immunodeficient recipients (Morrison and Weissman, 1994, Spangrude et al., 1988, Weissman and Shizuru, 2008). Mutations accumulating in HSPCs contribute to numerous blood disorders. Precise gene editing in HSPCs is a powerful tool for studying the functional consequences of such mutations.

The CRISPR/Cas9 system is commonly used for precise gene editing. In this system, single-guide RNAs (sgRNAs) guide Cas9 nucleases to the target sequence, where they induce DNA double-strand breaks (DSBs). These DSBs are predominantly repaired by the non-homologous end joining (NHEJ) pathway, causing micro-insertions or micro-deletions (indels). In the presence of a donor DNA template, the homologous recombination (HR) pathway is used to a lesser extent to precisely replace the damaged DNA sequence (Chu et al., 2015, Cong et al., 2013, Hsu et al., 2013, Mali et al., 2013). Previously, the delivery of the CRISPR/Cas9 system as preassembled ribonucleoprotein (RNP) complexes of Cas9 proteins and synthetic sgRNAs was shown to lead to high knockout frequencies in human and mouse primary HSPCs (Gundry et al., 2016). The combination of such RNPs with recombinant adeno-associated virus (rAAV) serotype 6 donor templates resulted in high frequencies of HR in human CD34^+^ HSPCs (Bak et al., 2017, Bak et al., 2018, De Ravin et al., 2016, Dever et al., 2016, Kuo et al., 2018, Sather et al., 2015). However, efficient CRISPR/Cas9-mediated knockin strategies in mouse HSPCs have not been reported.

The adeno-associated virus (AAV) genome is a single-stranded DNA of ∼4.700 nucleotides encoding replication and capsid genes that are flanked by 145-bp-long inverted terminal repeats. Natural variants and engineered serotypes have differential tissue tropism. They vary in their transduction efficiency, depending on the tissue or cell type (Gonçalves, 2005, Grimm et al., 2008, Russell and Hirata, 1998). An AAV serotype (AAV-DJ) was generated by gene shuffling technology, containing a hybrid capsid from 8 AAV serotypes. Despite shared characteristics with its closest relative AAV serotype 2 (AAV2), AAV-DJ has a broader and higher transduction efficiency than AAV2 (Grimm et al., 2008, Lerch et al., 2012).

Here, we describe a workflow to achieve efficient HR and gene repair in mouse HSPCs using CRISPR/Cas9 RNPs and AAV-DJ viruses for the delivery of donor templates. Using a fluorescent reporter system, we achieved high HR efficiency in the Lamin B1 (*Lmnb1*) and β-actin (*Actb*) loci. Furthermore, we confirmed that this method did not lead to detectable off-target effects in mouse HSPCs when an sgRNA with high specificity was used. The CRISPR/Cas9-edited mouse HSPCs contributed to all immune cell lineages upon transplantation into immunodeficient recipients. Using a similar approach, we repaired the neomycin-disrupted *Rag2* gene in HSPCs isolated from Rag2^−/−^ mice. The Rag2-corrected HSPCs engrafted into the immunodeficient recipients and reconstituted B and T cells. Altogether, we describe a method to study gene function and model human hematological diseases through *ex vivo* genetic manipulation of mouse HSPCs.

## Results

### CRISPR/Cas9-Mediated Gene Insertion in Mouse Cas9 Transgenic HSPCs

**Figure 1:**
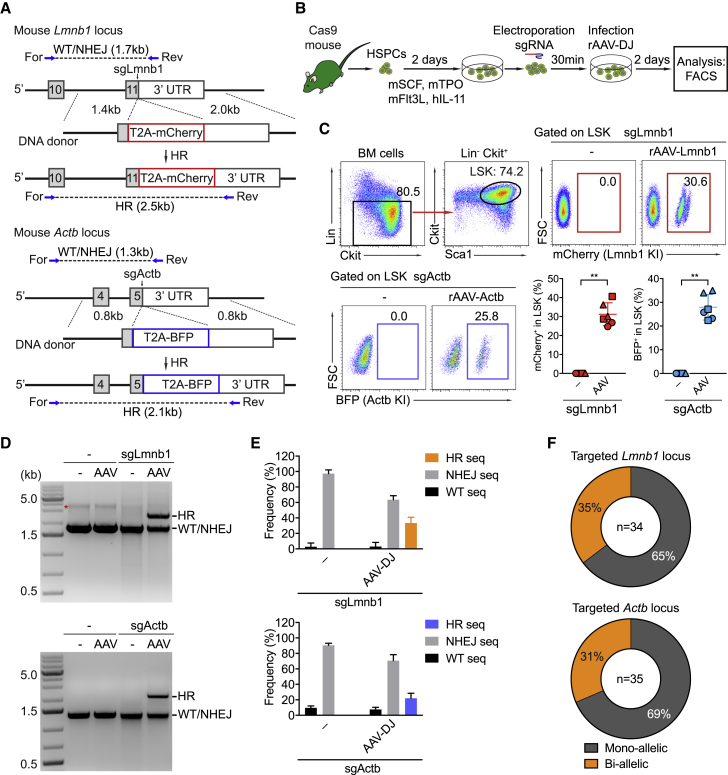
CRISPR/Cas9-Mediated Gene Insertion in Mouse Cas9 Transgenic HSPCs (A) Targeting strategy to insert T2A-mCherry and T2A-BFP into the *Lmnb1* and *Actb* loci, respectively. (B) Experimental scheme of CRISPR/Cas9-mediated gene insertion in mouse HSPCs. (C) FACS analysis of the targeted LSK cells 3 days after targeting *in vitro*. Gates show the percentages of mCherry^+^ or BFP^+^ LSK cells, based on the indicated gating strategy (top left). Circles, squares, and triangles represent individual mice (n = 6) from three independent experiments. Data are shown as means ± SD (^∗∗^p < 0.01, Mann-Whitney test). (D) HR and wild-type (WT) and/or NHEJ fragments were amplified by PCR, using the primers indicated in (A), in control or targeted LSK cells. (E) Frequencies of HR and NHEJ events in targeted HSPCs that received either sgRNA only or sgRNA and AAV-DJ donor vectors for the *Lmnb1* loci (top) and *Actb* loci (bottom). (F) Pie charts summarizing the frequency of monoallelic and bi-allelic HR events in the targeted *Lmnb1* loci (top) and *Actb* loci (bottom) of individual reporter^+^ colonies (see also Figure S2B). Data are based on at least three independent experiments. KI, knockin.

To quantitatively determine HR efficiency in mouse HSPCs, we aimed to insert the coding sequences of the self-cleavage peptide coupled to the fluorescent markers mCherry and BFP in frame into the last exon of mouse *Lmnb1* and *Actb*, respectively (Figure 1A). First, we used CrispRGold to design a highly specific sgRNA to target the *Lmnb1* gene near the STOP codon. To target the *Actb* locus, we chose a published sgRNA (Yao et al., 2017). These two sgRNAs are termed sgLmnb1 and sgActb hereafter. To test the gene editing activity of sgLmnb1 and sgActb, Sca1^+^ HSPCs isolated from Cas9-transgenic mice (Cas9-HSPCs) were activated with mouse SCF, TPO, and Flt3L and human interleukin-11 (IL-11) for 2 days and then electroporated with sgLmnb1 and sgActb (Figure 1B). Based on sequencing data of the targeted loci, both sgRNAs led to efficient gene editing with approximately 80% of frameshift mutations (Figures S1A and S1B). To assess HR efficiency, the activated Cas9-HSPCs were electroporated with either sgLmnb1 or sgActb and subsequently infected with AAV-DJ vectors carrying the corresponding donor templates for Lmnb1 (AAV-DJ-Lmnb1) or Actb (AAV-DJ-Actb) at a multiplicity of infection (MOI) of 5 × 10^6^ virus genome copies (GCs) per cell (Figure 1B). Three days after targeting, the edited HSPCs were analyzed by flow cytometry to quantitatively determine HR efficiency. Within the Lin^−^Sca1^+^cKit^+^ (LSK) cell compartment, we detected 31% ± 5% mCherry^+^ and 28% ± 5% BFP^+^ cells in the experimental groups receiving both sgRNA and AAV-DJ template vectors for *Lmnb1* and *Actb* loci, respectively (Figure 1C). To genetically confirm the HR events in the targeted loci, we amplified the targeted sites from gDNA of untreated and treated HSPCs using an external forward primer, annealing to a genomic sequence outside of the 5′ homology arm, and a reverse primer inside the targeted sequence (Figure 1A). As predicted, the targeted fragments of 2.5 kb (in the case of Lmnb1) and 2.1 kb (in the case of Actb) were amplified from the HSPCs that received the respective sgRNAs and AAV-DJ donor template vectors (Figure 1D; Figure S1C). Next, we cloned and sequenced PCR products of the targeted loci to quantify HR and NHEJ frequencies. Mouse HSPCs that were only treated with sgRNAs showed NHEJ events in 90% of the cases, of which 80% caused frameshift mutations. In the presence of AAV-DJ donor vectors, HR levels were 34% and 23% for the *Lmnb1* and *Actb* loci, respectively (Figure 1E; Figure S2A). To determine the frequency of mono- or bi-allelic gene knockin, we performed colony-forming assays by sorting the reporter^+^ LSK cells 2 days after targeting. The zygosity of the colonies was then analyzed by PCR. We detected bi-allelic gene knockin in 35% (for Lmnb1) and 31% (for Actb) of the reporter-positive HSPCs (Figure 1F; Figure S2B). Thus, using Cas9-transgenic mice, synthetic modified sgRNAs, reduced degradation by nucleases (Yin et al., 2017), and rAAV-DJ as a template delivery system, we achieved efficient HR-mediated gene knockin in mouse HSPCs.

### Off-Target Analysis of sgLmnb1 and sgActb in Mouse HSPCs

**Figure 2:**
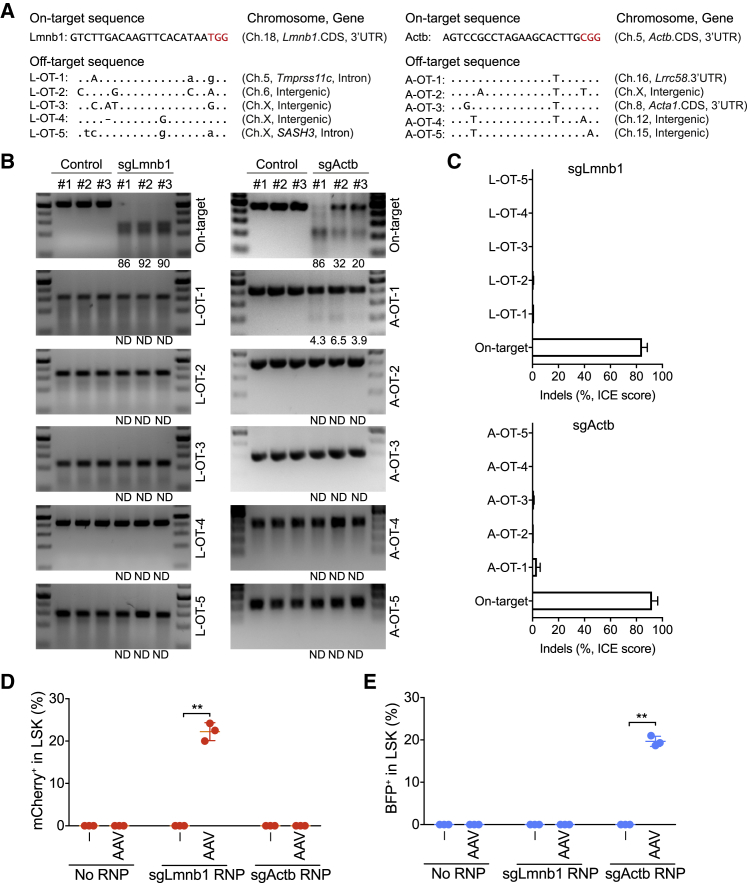
Off-Target Analysis in Mouse HSPCs (A) On-target and 5 highest-risk off-target sites of sgLmnb1 and sgActb as predicted by CrispRGold. (B) T7EI assays show editing activity of sgLmnb1 (left) and sgActb (right) in the on- and/or off-target sites from control or treated HSPCs (ND, not determined). (C) ICE analysis showing the indel frequency in the on- and/or off-targeted sites. Data are shown as means ± SD from three independent experiments. (D and E) Activated-Sca1^+^ HSPCs from C57BL/6 mice were electroporated with RNPs containing the indicated sgRNAs and infected with the indicated donor viruses. The frequency of mCherry^+^ (D) and BFP^+^ (E) LSK cells was determined by flow cytometry based on three independent experiments. Data are shown as means ± SD (^∗∗^p < 0.01, Mann-Whitney test).

Although CRISPR/Cas9 is a powerful tool for genome engineering, unwanted off-target activity remains a concern (Cradick et al., 2013, Elms et al., 2013, Frock et al., 2015, Pattanayak et al., 2013, Tsai and Joung, 2016). It has been reported that highly specific sgRNAs have less or no off-target activity in mouse liver (Akcakaya et al., 2018). To detect potential off-target editing events by CRISPR/Cas9 in mouse HSPCs, we used CrispRGold to predict specificity and the top 5 potential off-target sites of sgLmnb1 and sgActb. sgLmnb1 does not possess predicted high-risk off-targets, in agreement with specificity being a key criterion of CrispRGold. In contrast, the predicted specificity of sgActb, published by others and not designed by CrispRGold, was low, with one potential off-target site having only a single mismatch (Figure 2A). To assess the actual off-target editing, we amplified the top 5 off-target sites of sgLmnb1 and sgActb in the targeted HSPCs. We first estimated the indel frequencies at the on-target and off-target sites using the T7 endonuclease I (T7EI) assay. Based on this assay, 90% of the sequences of the sgLmnb1 site underwent NHEJ, whereas none of the 5 off-target sites led to a detectable cleavage product. In contrast, the top predicted off-target of sgActb (A-OT-1) led to a weak but detectable band, suggesting that NHEJ had occurred in 3%–6% of these sequences (Figure 2B). To confirm these data, we performed amplicon sequence deconvolution using the inference of CRISPR edit (ICE) sequencing analysis tool (Synthego) (Hsiau et al., 2019). According to this analysis, NHEJ occurred at high frequencies at the target sites of sgLmnb1 and sgActb. Consistent with the T7EI assay, sgLmnb1 led to no detectable off-target cleavage in all 5 off-target sites, whereas sgActb created a substitution in a few percent of the sequences of A-OT-1 (Figure 2C; Figure S3). Thus, in line with the previous publication, our data indicate that off-target activities can be avoided by the use of highly specific sgRNAs (Akcakaya et al., 2018). *In silico* prediction of off-target analysis is quick and simple, but with less sensitivity and limited off-target sites. To overcome the limitations of *in silico* prediction, an unbiased, genome-wide off-target analysis must be considered.

### CRISPR/Cas9-Mediated Gene Insertion in Wild-Type HSPCs

Next, we assessed whether we can achieve efficient HR in mouse HSPCs isolated from wild-type animals. Sca1^+^ HSPCs were isolated from C57BL/6 mice and activated as described (Figure 1B). These cells were then electroporated with sgLmnb1/Cas9 or sgActb/Cas9 RNP complexes and subsequently infected with rAAV-DJ-Lmnb1 or rAAV-DJ-Actb donor vectors, respectively. Consistent with the previous findings, we detected 22% ± 2% of mCherry^+^ LSK cells (in the case of Lmnb1) and 20% ± 1% of BFP^+^ LSK cells (in the case of Actb) 3 days after targeting (Figures 2D and 2E; Figure S4). To control for random integrations of the donor template into potential off-target Cas9-induced DSBs, we introduced DSBs in the *Lmnb1* locus by sgLmnb1/Cas9 RNPs but then infected the cells with the donor vector specific for Actb (rAAV-DJ-Actb), and vice versa, for the *Actb* locus. In both cases, we did not detect reporter-positive cells that would suggest integrations into the wrong locus (Figures 2D and 2E; Figure S4). These data indicate that under the given experimental conditions, reporter expression reflects correct locus-specific gene targeting, rather than unspecific integration into off-target sites.

### Titration of AAV-DJ and sgRNA for Gene Knockin in Mouse HSPCs

**Figure 3:**
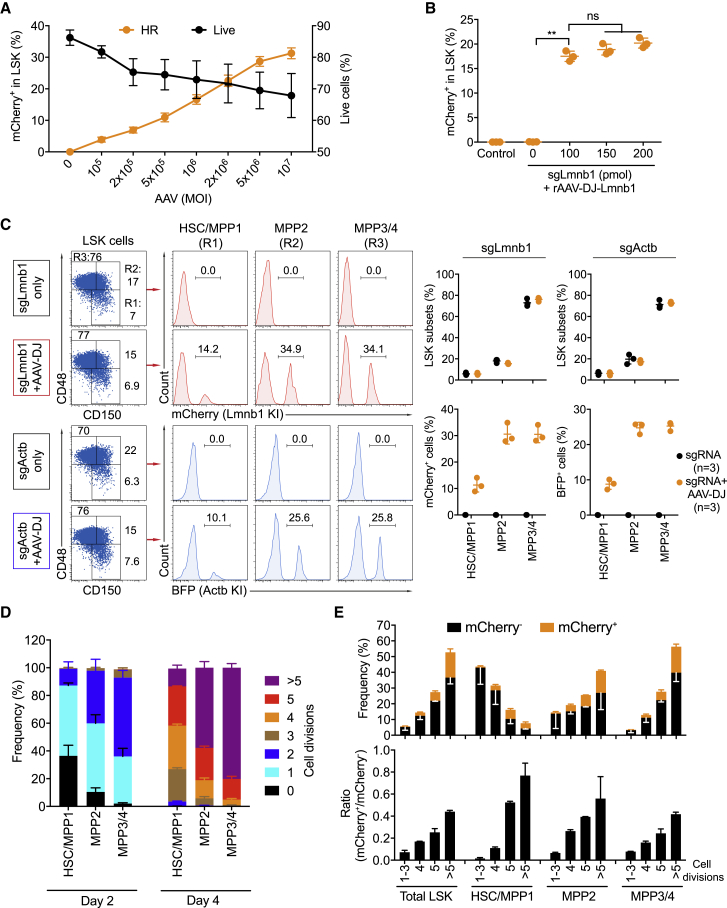
Titration of AAV-DJ Vectors, sgRNA, and HR Efficiency in Mouse LSK Subsets (A) HR efficiency and survival of activated Cas9-HSPCs that were electroporated with sgLmnb1 and infected with rAAV-DJ-Lmnb1 viruses at the indicated MOIs. Data are based on three independent experiments. (B) HR efficiencies in stimulated Cas9-HSPCs that were electroporated with the indicated doses of sgLmnb1 and infected with rAAV-DJ-Lmnb1 donor vectors at an MOI of 2 × 10^6^ GC/cell. The percentages of mCherry^+^ LSK cells are shown. Data are shown as means ± SD from three independent experiments (^∗∗^p < 0.01, Mann-Whitney test); ns, not significant (p > 0.05). (C) FACS analysis showing frequency of mCherry^+^ cells (for Lmnb1) and BFP^+^ cells (for Actb) within HSC and MPP1 (R1), MPP2 (R2), and MPP3/4 (R3) LSK subsets receiving either sgRNA only or sgRNA and AAV-DJ donor vectors. Graphs summarize the percentages of LSK subsets and frequency of reporter^+^ cells in the HSC and MPP1, MPP2, and MPP3/4 subpopulations from three independent experiments. (D) Percentage of cell division in each LSK subset at days 2 and 4 after CellTrace labeling. (E) Frequency and ratio of mCherry^+^ and mCherry^−^ cells of LSK subsets in dependence of cell division. Data are shown as means ± SD from three independent experiments.

AAV serotype 6 (AAV6)-mediated template delivery was reported to support efficient HR in human CD34^+^ HSPCs with viral titers as low as 10^5^ GC/cell (Bak et al., 2018, Bak and Porteus, 2017, Dever et al., 2016). In contrast to the human data, the transduction efficiency of AAV6 vectors in mouse HSPCs was reported to be highly variable (Ellis et al., 2013, Song et al., 2013). To compare the HR efficiencies mediated by AAV6 and AAV-DJ as donor template vectors side by side, stimulated Cas9-HSPCs were electroporated with either sgLmnb1 or sgActb and infected with AAV6 or AAV-DJ at an MOI of 1 × 10^6^ GC/cell. AAV-DJ allowed higher HR efficiencies than AAV6 in our system (Figure S5A). To determine the optimal dose of rAAV-DJ vectors for efficient HR-mediated gene editing in mouse HSPCs, we performed rAAV titrations. Stimulated Cas9-HSPCs were electroporated with sgLmnb1 and subsequently infected with rAAV-DJ-Lmnb1 vectors at increasing MOI. As expected, the MOI correlated with the HR efficiency detected 3 days after targeting. Consistent with data on human CD34^+^ HSPCs, the survival of the edited mouse HSPCs decreased at high MOI (Figure 3A; Figure S5B) (Bak and Porteus, 2017, Schiroli et al., 2019). Altogether, an AAV-DJ dose of 5 × 10^6^ GC/cell allows high efficiency of gene knockin at an acceptable low toxicity.

High HR-mediated gene knockin frequencies require efficient DSB introduction by CRISPR/Cas9 (Liang et al., 2017). Because Cas9 is constitutively expressed in the Cas9-HSPCs we used, we reasoned that increasing amounts of sgRNAs might elevate HR efficiency. To test this hypothesis, stimulated Cas9-HSPCs were electroporated with different doses of sgLmnb1 or sgActb and subsequently infected with rAAV-DJ-Lmnb1 or rAAV-DJ-Actb vectors at an MOI of 2 × 10^6^ GC/cell. Based on the frequency of mCherry^+^ or BFP^+^ cells detected 3 days after targeting, 100 pmol of sgRNA represents an optimal dose for gene knockin in mouse HSPCs (Figure 3B; Figure S5C).

### HR Efficiency within Cas9-Expressing Mouse LSK Subsets

LSK cells are a heterogeneous population, including HSCs and multipotent progenitor (MPP) subsets with decreasing reconstitution potential. MPPs can be classified into the MPP1, MPP2, MPP3, and MPP4 subpopulations based on the expression of signaling lymphocytic activation molecule (SLAM) markers, such as CD48 and CD150 (Cabezas-Wallscheid et al., 2014, Kiel et al., 2005, Pietras et al., 2015). CD48^−^CD150^+^ LSK cells include HSCs and MPP1. CD48^+^CD150^+^ and CD48^+^CD150^−^ LSK cells include the cell lineage-restricted MPP2 and MPP3/4 subsets, respectively. To quantify the HR efficiency in each LSK subpopulation, we analyzed the frequencies of reporter knockin in each subset 2 days after targeting *in vitro*. Within the MPP2 and MPP3/4 LSK subsets, 25%–30% of reporter-positive cells were detected in the cells receiving both sgRNA and AAV-DJ donor vectors. The HR efficiency in the HSC and MPP1 subset was only 8%–10% (Figure 3C). This may be related to the HR pathway being exclusively active in the S and G2 phases of the cell cycle (Branzei and Foiani, 2008, Pietras et al., 2011, Sonoda et al., 2006). To investigate whether HR efficiency is correlating with cell proliferation in our system, we measured the proliferation rates of these LSK subsets using CellTrace labeling. The labeled LSK cells were analyzed at days 2 and 4 by flow cytometry. In line with previous publications, cell proliferation rates in the HSC and MPP1 2 and 4 days after stimulation were significantly lower than in the MPP2 and MPP3/4 subsets (Figure 3D; Figure S6A) (Pietras et al., 2015, Wilson et al., 2008). To correlate HR efficiency and cell division for each LSK subset, we labeled the cells with CellTrace and performed the knockin experiments as before. AAV-DJ infection did not affect proliferation (Figure S6B). The percentage of mCherry^+^ cells correlated with the number of cell divisions in all LSK subsets, with apparently no mCherry^+^ cells in undivided cells (Figure 3E; Figure S6C). Thus, our data confirm that HR efficiency depends on the cell cycle and show that our method allows HR in about 10% of the HSC and MPP1 population in Cas9-expressing mice.

### Testing of Donor Template Types for Gene Insertion in Mouse HSPCs

**Figure 4:**
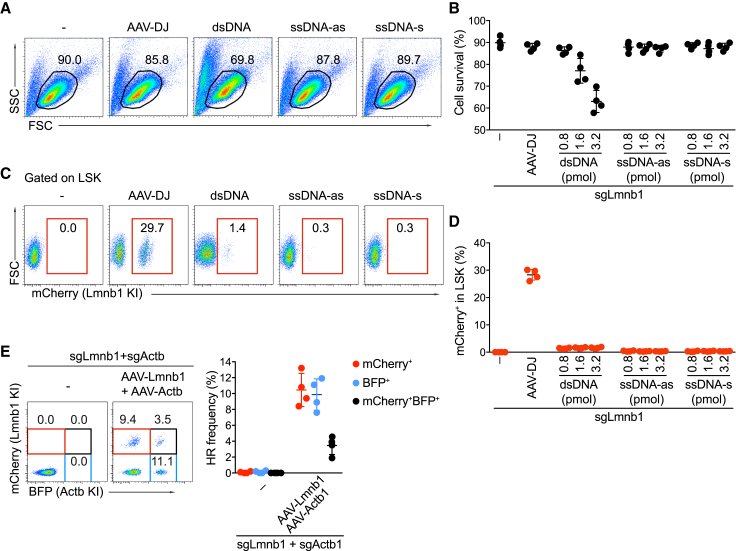
Non-Viral DNA Donor Templates Are Not Suitable for Efficient Gene Insertion in Mouse HSPCs (A) Survival of Cas9-HSPCs that were electroporated with sgLmnb1 and treated with the indicated types of non-viral donor templates: dsDNA and ssDNA antisense (as) or sense (s), based on flow cytometry. As a positive control, rAAV-DJ-Lmnb1 donor viruses were used at an MOI of 5 × 10^6^ GC/cell. Numbers indicate the percentage of live cells. (B) Summary of the data shown in (A) from four independent experiments. (C) FACS analysis showing the percentages of mCherry^+^ LSK cells in the various groups. (D) Summary of (C) based on four independent experiments. (E) mCherry and BFP knockin frequencies in Cas9-HSPCs that were electroporated with sgLmnb1 and sgActb and infected with the indicated donor viruses (each at a 5 × 10^6^ GC/cell). The gates are set on LSK cells positive for mCherry (red), BFP (light blue), and double reporter (black). The graph (right) summarizes knockin efficiencies from four independent experiments.

Double- and single-stranded DNA (dsDNA and ssDNA) templates were successfully used as donor templates for Cas9-mediated HR in human primary T cells and HSPCs (DeWitt et al., 2016, Gundry et al., 2016, Roth et al., 2018, Schumann et al., 2015). In comparison to AAV-DJ, the generation of dsDNA and ssDNA templates is technically simple, prompting us to test whether these templates are compatible with high HR efficiency in mouse HSPCs. To this end, dsDNA and ssDNA (sense and antisense) templates were generated using complete and nicking restriction enzyme digestion, respectively (Figure S7). These were then used at increasing doses to insert mCherry into the *Lmnb1* locus of Cas9-HSPCs. In these experiments, the dsDNA donor template led to a clear reduction of cell survival, suggesting that dsDNA templates are toxic to mouse HSPCs. In contrast, the ssDNA donor template did not affect cell viability, even at the highest concentration used (3.2 pmol) (Figures 4A and 4B). However, both dsDNA and ssDNA donor templates led to low HR frequencies below 2% (Figures 4C and 4D). Thus, dsDNA and ssDNA donor templates appear unsuitable for efficient Cas9-mediated gene insertion in mouse HSPCs.

### Simultaneous Gene Insertions in Two Loci in Mouse HSPCs

Many hematological disorders are caused by mutations in two or even more genes. The generation of transgenic mouse models with two or more mutations is challenging and time consuming. We reasoned that the insertion of mutations in two or more genes of mouse HSPCs *in vitro* would significantly facilitate the modeling of genetically complex hematological diseases. We thus established multiplex gene insertions into mouse HSPCs by simultaneously electroporating sgLmnb1 and sgActb, followed by infection with both the AAV-DJ-Lmnb1 and the AAV-DJ-Actb donor vectors (each at an MOI of 5 × 10^6^ GC/cell). This approach led to 4% ± 1% of LSK cells that were double positive for mCherry and BFP 3 days after targeting, in addition to the mCherry^+^ (10% ± 2%) and BFP^+^ (10% ± 2%) single-positive cells (Figure 4E). These data show that our targeting system enables simultaneous gene insertion or editing at two loci of mouse LSK cells.

### The Targeted HSPCs Repopulate All Immune Cell Lineages

**Figure 5:**
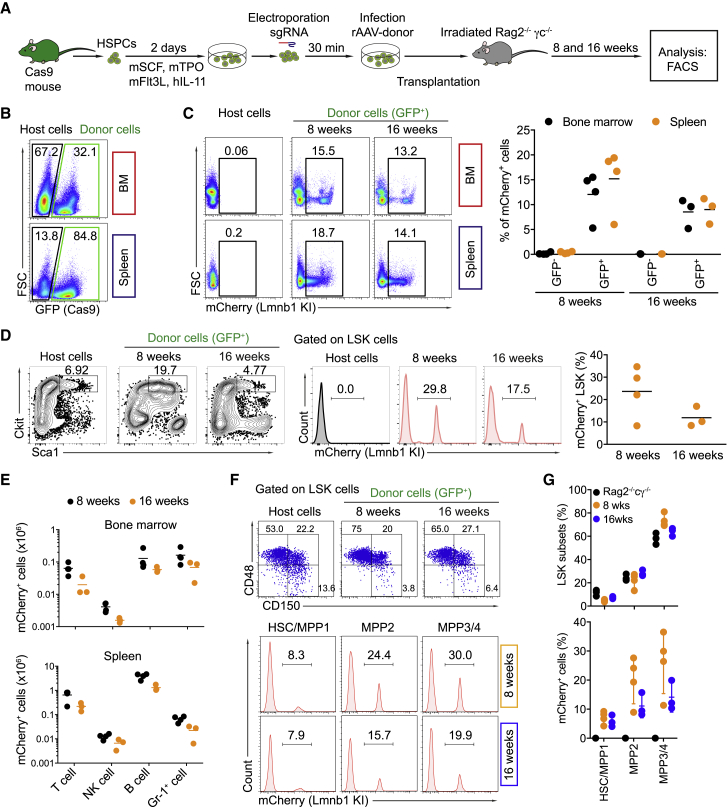
Targeted HSPCs Repopulate the Immune Cell Lineages (A) Experimental scheme of mCherry insertion into the *Lmnb1* gene in HSPCs that were transplanted into irradiated Rag2^−/−^c_γ_^−/−^ mice. Hematopoietic cell lineages were analyzed by flow cytometry 8 weeks (n = 4) or 16 weeks (n = 3) after reconstitution. (B) FACS analysis showing the percentages of donor cells (GFP^+^) in the bone marrow (BM) and spleen of the recipient mice 8 weeks after reconstitution. (C) Frequency of mCherry^+^ cells among GFP^−^ (host) or GFP^+^ (donor) cells in the bone marrow and spleen of the recipients determined by flow cytometry (left). The graph depicts percentages of mCherry^+^ cells in the bone marrow (black) and spleen (orange) of the recipient animals 8 weeks (n = 4) and 16 weeks (n = 3) after reconstitution. (D) LSK gating (left) and frequency of the mCherry^+^ cells among the LSK cells 8 and 16 weeks after reconstitution (right). The graph (right) shows the data from 4 recipient animals (8 weeks) or 3 recipient animals (16 weeks). (E) Absolute numbers of mCherry^+^ T, B, NK, and Gr-1^+^ cells in the bone marrow and spleen of the recipient animals 8 weeks (black) or 16 weeks (orange) after reconstitution. (F) FACS analysis showing frequencies of mCherry^+^ cells among the HSC/MPP1, MPP2, and MPP3/4 LSK subpopulations in the bone marrow of the recipient mice 8 and 16 weeks after reconstitution. (G) Graphs depict percentages of LSK subsets (top) and percentages of mCherry^+^ cells within each subset (bottom).

To verify that the CRISPR/Cas9-targeted mouse HSPCs maintained long-term engraftment and multi-lineage differentiation capacity, we transplanted the Lmnb1-targeted LSK cells into sublethally irradiated Rag2^−/−^cγ^−/−^ recipient mice and analyzed hematopoietic reconstitution 8 and 16 weeks after transplantation (Figure 5A). 8 weeks after transplantation, 26% ± 3% and 74% ± 7% of bone marrow and splenic cells, respectively, were GFP^+^ and thus donor derived. After 16 weeks, the percentages decreased to 17% ± 8% in the bone marrow but remained constant in the spleen (74% ± 4%) (Figure 5B; Figure S8A). To test whether AAV-DJ infection affects the reconstitution potential of LSK cells, we transplanted the targeted LSK cells that received either sgLmnb1 only or both sgLmnb1 and AAV-DJ donor vectors (5 × 10^6^ GC/cell) into irradiated Rag2^−/−^cγ^−/−^ recipient mice. AAV-DJ transduction resulted in some reduction, but not a major reduction, of engraftment efficiency (Figure S8E).

8 weeks after reconstitution, 14% ± 1% and 18% ± 1% of the GFP^+^ cells were mCherry^+^ in the bone marrow and spleen, respectively. The percentages of mCherry^+^ cells decreased to 10% ± 0.6% in the bone marrow and 10% ± 0.8% in the spleen 16 weeks after reconstitution (Figure 5C). This overall decrease in mCherry^+^ cells was consistent with a reduction of mCherry^+^ LSK cells in the bone marrow of the recipients at the later time point (Figure 5D). Moreover, the percentages and absolute cell numbers of mCherry^+^ T, B, natural killer (NK), and granulocytes in both bone marrow and spleen of the recipients were lower at 16 weeks compared with 8 weeks after the reconstitution (Figure 5E; Figures S8B–S8D). The reduction of mCherry^+^ cells in the recipient animals 16 weeks after reconstitution suggested that most transplanted mCherry^+^ LSK cells were cell lineage-committed progenitor cells that provided only short-term reconstitution (Figure 3C). To test this hypothesis, we analyzed the frequency of mCherry^+^ cells within the LSK subpopulations of the recipient animals 8 and 16 weeks after reconstitution. Although in the MPP2 and MPP3/4 subsets the percentage of mCherry^+^ cells was reduced at week 16, the percentage of mCherry^+^ cells within the HSC and MPP1 LSK population was stable around 6% from 8 to 16 weeks after reconstitution (Figures 5F and 5G). Collectively, the targeted HSPCs were able to engraft and repopulate all immune cell lineages in the recipient animals 5 weeks after secondary reconstitution, with a percentage of around 5%–6% (Figure S9). Overall, our data show that the edited HSPCs engraft and repopulate all immune lineages upon transplantation into immunodeficient mice.

### Repair of a Rag2 Knockout Allele

**Figure 6:**
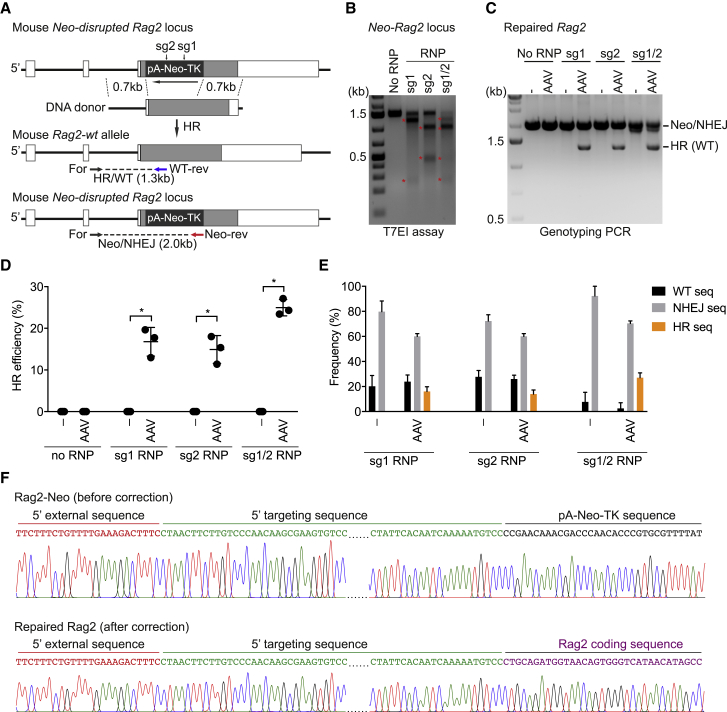
Repair of a Neomycin-Disrupted Rag2 Gene in Mouse HSPCs (A) Targeting strategy of CRISPR/Cas9-mediated correction of the neomycin-disrupted *Rag2* gene. A PCR strategy amplifies HR and NHEJ events based on the indicated primers. (B) T7EI assay on activated Sca1^+^ HSPCs from Rag2^−/−^ mice that were electroporated with RNPs containing the indicated sgRNAs. Stars indicate the cleaved fragments. (C) PCR amplification of Neo (without or with indels, Neo/NHEJ) and the repaired *Rag2* gene (WT) in HSPCs that were treated as indicated, based on the primers shown in (A). (D) Quantification of HR efficiency in (C). Data are shown as means ± SD (^∗^p < 0.05, Mann-Whitney test) from three independent experiments. (E) Frequencies of HR and NHEJ events in targeted HSPCs that received either RNP only or RNP and AAV-DJ donor vectors based on three independent experiments. (F) Sanger sequencing data showing the junctions of the 5′ homology arm of the neomycin-disrupted *Rag2* (before correction, top) and the repaired *Rag2* locus (after correction, bottom). Data are based on at least three independent experiments.

Next, we sought to repair the disrupted *Rag2* gene in Rag2-deficient HSPCs, derived from *Rag2* knockout animals, by CRISPR/Cas9. Rag2 knockout mice had previously been generated by inserting a TK-Neo-pA cassette into the coding sequence of exon 2 of the *Rag2* gene (Shinkai et al., 1992). We designed two sgRNAs targeting the TK-Neo-pA cassette, termed sg1 and sg2 hereafter (Figure 6A). Both sgRNAs showed high editing activity, based on T7EI assays, after electroporation of the sgRNAs within RNPs into Sca1^+^ HSPCs isolated from Rag2^−/−^ mice (Figure 6B). To repair the disrupted *Rag2* allele, we electroporated Sca1^+^ HSPCs with RNPs containing both sgRNAs and, 30 min later, infected them with AAV-DJ vectors carrying the wild-type Rag2 (AAV-DJ-Rag2) donor template. Three days after targeting, the efficiency of HR-mediated repair in the *Rag2* locus was assessed by PCR, using the primer sets indicated in Figure 6A. Although Neo-disrupted *Rag2* alleles (with or without indels, termed Neo/NHEJ) were detectable in all conditions, the repaired Rag2 fragment (1.3 kb) was only present in mouse HSPCs that received both sgRNA-RNP and AAV-DJ-Rag2 donor vectors (Figure 6C). Band quantification and sequencing data indicated that gene repair occurred in approximately 14% to 25% of HSPCs treated with the AAV-DJ donor vector and RNPs sg1, sg2, or sg1+sg2 (Figures 6D and 6E; Figure S10). Sanger sequencing confirmed the successful repair of the mutant (Neo-disrupted) *Rag2* allele (Figure 6F).

**Figure 7:**
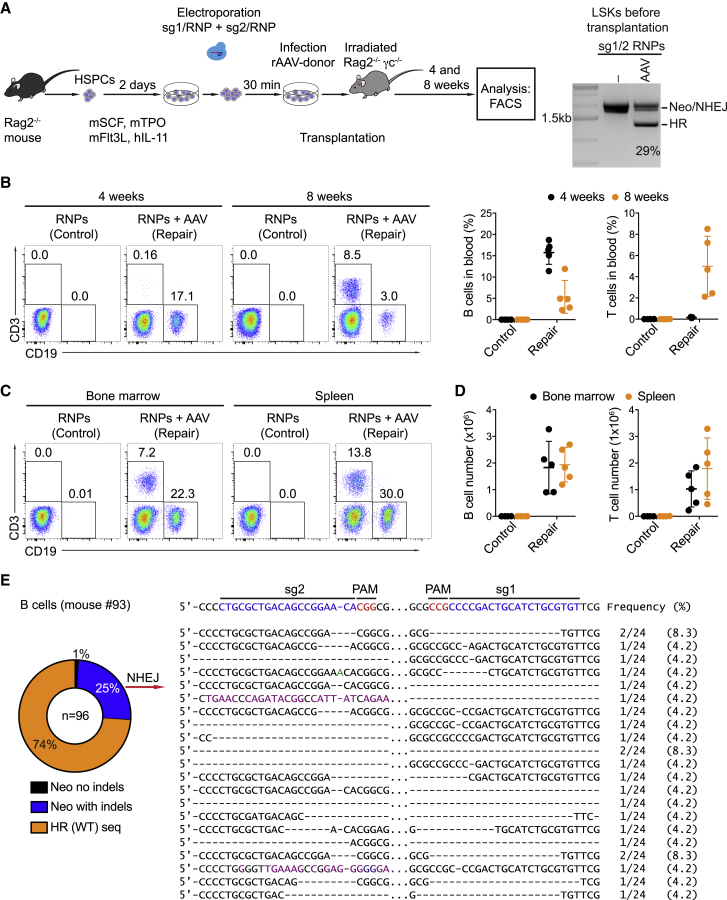
HSPCs with CRISPR/Cas9-Repaired Rag2 Restore B and T Cell Development *In Vivo* (A) Experimental scheme of CRISPR/Cas9-mediated Rag2 correction in HSPCs from Rag2^−/−^ mice. The targeted HSPCs were then transplanted into irradiated Rag2^−/−^cγ^−/−^ mice, which were analyzed 4 and 8 weeks after reconstitution. HSPCs treated with RNPs only were transplanted as controls. HR efficiency was verified in the edited HSPCs before transplantation using the PCR strategy indicated in Figure 6A. (B) Percentages of CD19^+^ B and CD3^+^ T cells in the blood of representative control recipients (n = 4) and experimental recipients (n = 5, termed repair), as determined by flow cytometry (left), and summary of the data (right; dots represent individual mice). (C) FACS analysis showing the percentages of B and T cells in the bone marrow and spleen of the recipients 8 weeks after reconstitution. (D) Numbers of B and T cells in the bone marrow (black) and spleen (orange) of control and repair groups 8 weeks after transplantation. (E) Sequencing data showing frequencies of HR (WT) and NHEJ events in B cells from a recipient mouse 8 weeks after reconstitution. The sequences of the NHEJ events reveal diversity (right).

In a next step, we assessed whether the repaired Rag2 gene was functional. Rag2 is critical for B cell receptor (BCR) and T cell receptor (TCR) rearrangements. As a consequence, the development of B and T cells is blocked in the Rag2^−/−^ mice (Shinkai et al., 1992). To see whether the Rag2-corrected mouse HSPCs can rescue B cell and T cell development, we transplanted Rag2-repaired Sca1^+^ HSPCs into Rag2^−/−^cγ^−/−^ recipient animals 2 days after repair. As controls, we transplanted non-repaired cells that were treated with RNPs, but not infected with the repair template, into separate recipients. Before transplantation, approximately 29% of the targeted HSPCs showed successful repair of the Neo-disrupted *Rag2* allele (Figure 7A). We then analyzed the presence of B and T cells in the peripheral blood of the recipient animals 4 and 8 weeks after reconstitution. A substantial population of B cells was present in the blood of the recipient animals that received the repaired HSPCs after 4 weeks (17%), and a detectable population of T cells (8%) became detectable after 8 weeks of reconstitution (Figure 7B). Consistent with these observations, 8 weeks after transplantation, B and T cells were present in the bone marrow and the spleen of the animals that were reconstituted with the repaired HSPCs (Figures 7C and 7D). To unbiasedly quantify the HR efficiency *in vivo*, we determined HR levels in NK cells, a cell type known to develop independently of Rag2 (Shinkai et al., 1992). Using the amplification strategy for the repaired *Rag2* locus (Figure 6A), we observed 17% and 28% of HR in NK cells (Figure S11A). To exclude that the lymphocytes in the reconstituted mice originate from a few or a single Rag2-repaired HSC, we traced CRISPR/Cas9-induced scar mutations in the Neo-disrupted *Rag2* allele within monoallelically Rag2-repaired B cells of the transplanted animals (Figure 7E; Figures S11B and S11C). Scar mutations were highly diverse, indicating that B cells were derived from multiple monoallelically Rag2-corrected HSPCs. To assess whether Rag2-repaired HSPCs contribute to a secondary reconstitution, mixed LSK cells, including host (Rag2-deficient) and donor cells, from the transplanted animals 8 weeks after primary reconstitution, were transplanted into irradiated secondary recipients. 5 weeks after secondary reconstitution, B and T cells were detectable in the peripheral blood, bone marrow, and spleen of the animals (Figure S11D). Thus, using the CRISPR/Cas9 system, we were able to repair a neomycin-disrupted *Rag2* allele in mouse HSPCs and rescue B and T cell development.

## Discussion

Our work provides a protocol for efficient CRISPR/Cas9-mediated gene knock in and gene repair in mouse HSPCs. Our system allows 10% of HR in the HSC and MPP1 subset of mouse HSPCs, without detectable off-target activity at predicted off-target sites if a highly specific sgRNA is used. The edited HSPCs engrafted and repopulated all immune cell lineages upon transplantation into immunodeficient mice, an approach resembling bone marrow reconstitution in human patients after myeloablative conditioning (Bernardo and Aiuti, 2016). As a proof of principle, we successfully repaired a neomycin-disrupted Rag2 gene in mouse HSPCs. The repaired HSPCs regained functionality and gave rise to T and B cells in the recipient animals.

The CRISPR/Cas9 system holds promise for therapeutic applications (Cornu et al., 2017, Dunbar et al., 2018). However, many studies have shown that CRISPR/Cas9 exhibits off-target activity (Cradick et al., 2013, Elms et al., 2013, Frock et al., 2015, Pattanayak et al., 2013, Tsai and Joung, 2016). Akcakaya et al. (2018) provided evidence in an *in vivo* study that off-target effects can be minimized or avoided by the use of highly specific sgRNAs. In line with this observation, the highly specific sgLmnb1 did not lead to mutations in its predicted off-target sites in our study, whereas the less specific sgActb showed detectable cleavage at the off-target site with the highest predicted risk. Thus, to minimize unwanted off-target effects, sgRNAs have to be carefully designed to maximize the number of mismatches at the highest risk off-target sites (Bae et al., 2014, Chu et al., 2016a, Graf et al., 2019).

During the G1 phase, CRISPR/Cas9-induced DSBs are repaired preferentially by NHEJ, whereas cells in the S and G2 phases use the HR pathway (Branzei and Foiani, 2008, Sonoda et al., 2006). Thus, cellular proliferation rates are critical for maximizing HR efficiency in mouse HSPCs. Consistent with previous reports, we observed higher *in vitro* proliferation rates in the MPP2 and MPP3/4 LSK subsets compared with HSC and MPP1 (Passegué et al., 2005, Pietras et al., 2015). As a result, the HR efficiency in these subsets was significantly higher than in the long-term HSC and MPP1 subset. Similarly, HR levels in human long-term HSCs are significantly lower than those in MPP cells (Lomova et al., 2019, Pavel-Dinu et al., 2019). This is likely because human long-term HSCs are essentially non- or slowly dividing cells (Kim et al., 2014, Shin et al., 2018).

The efficiency of HR in mouse HSPCs is determined by the activity of CRISPR/Cas9 and the type and amount of donor template. We show that the delivery of Cas9 and sgRNAs by RNPs leads to efficient gene targeting in these cells, in line with a previous study (Gundry et al., 2016). By systematically testing the suitability of rAAV-DJ, dsDNA, and long ssDNA as donor vectors for HR in mouse HSPCs, we found that rAAV was the only donor template vector mediating efficient gene knockins. This was unexpected, because earlier studies had shown that dsDNA donor templates, as well as ssDNA donor templates, allow efficient HR-mediated gene modification in human primary T cells, human CD34^+^ HSPCs, and a range of human cell lines (Chu et al., 2015, DeWitt et al., 2016, Gundry et al., 2016, Kuo et al., 2018, Roth et al., 2018, Schumann et al., 2015). These differences might reflect differences in stability of DNA donor templates, DNA damage responses, DNA trafficking, and how well foreign DNA is tolerated by the cells.

Although AAV6 was successfully used for HR in human T cells and CD34^+^ HSPCs, it was reported to poorly infect mouse HSPCs (Ellis et al., 2013, Song et al., 2013). We show that AAV6 can be used as template delivery system for HR in mouse HSPCs but at a slightly lower efficiency compared with AAV-DJ vectors. A limitation of the AAV system is the maximal AAV packaging capacity of 4.5 kb. In these limits, we succeeded in the present study in inserting 0.8-kb reporters into the *Lmnb1* and *Actb* loci and replacing a neomycin cassette of 2 kb with a Rag2 wild-type sequence of 0.8 kb. Overall, we showed that the combination of CRISPR/Cas9 RNPs and rAAV-DJ donor templates represents an efficient system for targeted gene modification in mouse HSPCs.

Hematological diseases are caused by mutations in single or multiple genes (Corces-Zimmerman and Majeti, 2014, Ferraris et al., 1985, Fialkow et al., 1987, Miyamoto et al., 2000). The introduction of such mutations into mouse HSPCs represents a powerful tool to elucidate their *in vivo* functions in hematological disorders. However, generating such genetically engineered mouse models was hitherto time consuming and required conditional gene targeting in the mouse germline to restrict the mutation or mutations to cells of the hematopoietic system. By the simultaneous introduction of two targeted mutations into two genetic loci in HSPCs, we demonstrate that the present system is suitable to study hematopoietic diseases arising from multiple mutations.

## STAR★Methods

### Key Resources Table

**Table:** 

REAGENT or RESOURCE	SOURCE	IDENTIFIER
**Antibodies**		

PE anti-mouse Ly6A/E (Sca-1) (Clone D7)	BioLegend	Cat#108107: RRID:AB_313344
BV875 anti-mouse Ly6A/E (Sca-1) (Clone D7)	BioLegend	Cat#108139: RRID:AB_2565957
APC anti-mouse CD117 (c-kit) (Clone 2B8)	BioLegend	Cat#105812; RRID:AB_313221
BV605 anti-mouse CD150 (Clone TC15-12F12.2)	BioLegend	Cat#115927; RRID:AB_11204248
APC/Cy7 anti-mouse CD48 (Clone HM48-1)	BioLegend	Cat#103431: RRID:AB_2561462
PE/Cy7 anti-mouse B220 (Clone RA3-6B2)	BioLegend	Cat#103221: RRID:AB_313004
BV785 anti-mouse B220 (Clone RA3-6B2)	BioLegend	Cat#103245: RRID: AB_11203538
BV605 anti-mouse CD19 (Clone 6D5)	BioLegend	Cat#115539: RRID:AB_11203538
BV785 anti-mouse CD19 (Clone 6D5)	BioLegend	Cat#115543: RRID:AB_11218994
BV605 anti-mouse CD11b (Clone M1/70)	BioLegend	Cat#101237: RRID:AB_11126744
PE/Cy7 anti-mouse CD11b (Clone M1/70)	BioLegend	Cat#101215: RRID:AB_312798
Alexa Fluor® 700 anti-mouse Gr-1 (Clone RB6-8C5)	BioLegend	Cat#108421: RRID:AB_493728
PE anti-mouse Gr-1 (Clone RB6-8C5)	BioLegend	Cat# 108407: RRID:AB_313372
PE/Cy7 anti-mouse Gr-1 (Clone RB6-8C5)	BioLegend	Cat#108415: RRID:AB_313380
PE/Cy7 anti-mouse CD3e (Clone 145-2C11)	BioLegend	Cat#100319: RRID:AB_312684
Alexa Fluor® 647 anti-mouse CD3e (Clone 145-2C11)	BioLegend	Cat#100324: RRID:AB_492861
PE/Cy7 anti-mouse NK-1.1 (Clone PK136)	BioLegend	Cat#108713: RRID:AB_389363
PE/Cy7 anti-mouse TER-119 (Clone TER-119)	BioLegend	Cat#116221: RRID:AB_2137789
PE/Cy7 anti-mouse CD45 (Clone 30-F11)	BioLegend	Cat#103113: RRID:AB_312978
TruStain FcX (anti-mouse CD16/32, clone 93)	BioLegend	Cat#101320: RRID:AB_1574975

**Bacterial and Virus Strains**		

TOP10	Invitrogen	Cat#C404003
DH5α	Thermofisher	Cat#18265017
AAV-DJ	Cell Biolabs	Cat#VPK-400-DJ
AAV-6	Cell Biolabs	Cat#VPK-410-SER6

**Chemicals, Peptides, and Recombinant Proteins**		

Mouse recombinant SCF	Peprotech	Cat#250-03
Mouse recombinant TPO	Peprotech	Cat#315-14
Mouse recombinant Flt3-ligand	Peprotech	Cat#250-31L
Human recombinant IL-11	Peprotech	Cat#200-11
StemSpanTM SFEM II	Stemcell	Cat#09655
Gentamycin	Lonza	Cat#17-519L
spCas9	IDT	Cat#1074182
spCas9	MDC Berlin, Germany	N/A
Polyethylenimine (PEI)	Polysciences	Cat#23966-1
Pluronic F-68	Thermo Scientific	Cat#24040032
OptiPrepTM Density Gradient Medium	Sigma	Cat#D1556-250ML
Benzonase® endonuclease	Millipore	Cat#70746-3
DNase	QIAGEN	Cat#79254
DAPI	Sigma	Cat#D9542-5MG
AMPure XP beads	Beckman Coulter	Cat#A63881
RNA Gel Loading Dye (2x)	Thermo Scientific	Cat#R0641

**Critical Commercial Assays**		

Zero Blunt® TOPO® PCR Cloning Kit	Invitrogen	Cat#450245
TOPO® TA Cloning® Kit	Invitrogen	Cat# K4500-01
CloneJET PCR cloning kit	Thermo Scientific	Cat#K1232
Synthetic modified sgRNAs	Synthego	N/A
Alt-R® CRISPR sgRNAs	IDT	N/A
Alt-R® CRISPR tracrRNA	IDT	Cat#1072534
Anti-Sca-1 Microbead Kit (FITC), mouse	Miltenyi Biotec	Cat#130-092-529
TaqMan PCR master mix	Life technologies	Cat#4324018
NucleoSpin® Gel and PCR clean-up	Macherey-Nagel	Cat#740609.250
NucleoSpin® Plasmid	Macherey-Nagel	Cat#740588.250
Qiaquick® gel extraction kit	QIAGEN	Cat#28704
Herculase II Fusion DNA Polymerase	Agilent Technology	Cat# 600677
LongAmp® Taq 2X Master Mix	NEB	Cat# M0287L
CellTrace™ Violet Cell Proliferation Kit	Thermo Scientific	Cat#C34571
MethoCult GF M3434	Stemcell	Cat#03434
T7 endonuclease I assay	NEB	Cat# M0302S

**Experimental Models: Cell Lines**		

HEK293T	ATCC	ATCC®CRL-3216

**Experimental Models: Organisms/Strains**		

C57BL/6	Taconic	C57BL/6NTac
R26-Cas9iGFP	Chu et al., 2016b	N/A
Rag2^−/−^ cγ^−/−^	Taconic	N/A
Rag2^−/−^	Taconic	RAGN12

**Oligonucleotides**		

Synthetic modified sgLmnb1: 5′- GTCTTGACAAGTTCACATAA	This paper	N/A
Synthetic modified sgActb: 5′- AGTCCGCCTAGAAGCACTTG	Yao et al., 2017	N/A
Alt-R® CRISPR sg-1 to target the TK-Neo-pA: 5′- ACACGCAG ATGCAGTCGGGG	This paper	N/A
Alt-R® CRISPR sg-2 to target the TK-Neo-pA: 5′- CTGCGCTG ACAGCCGGAACA	This paper	N/A

**Recombinant DNA**		

pAAV-DJ	Cell Biolabs	Cat#VPK-420-DJ
pAAV-DJ-Lmnb1-T2A-mCherry	This paper	N/A
pAAV-DJ-Actb-T2A-BFP	This paper	N/A
pAAV-DJ-Rag2wildtype	This paper	N/A
pAAV-Helper	Cell Biolabs	Cat#VPK-420-DJ
pAAV-DJ-Rep/Cap	Cell Biolabs	Cat#VPK-420-DJ
pAAV-6-Rep/Cap	Cell Biolabs	# VPK-426
pTV-XhoI/NotI-Lmnb1-T2A-mCherry (dsDNA)	This paper	N/A
pTV-Nb.BsrDI/NotI-Lmnb1-T2A-mCherry (ssDNA)	This paper	N/A
pTV-Nb.BsrDI/NotI-Lmnb1-T2A-mCherry (asDNA)	This paper	N/A

**Software and Algorithms**		

Prism 7.0a	GraphPad	https://www.graphpad.com/
FlowJo 10.4.1	LLC	https://www.flowjo.com/
CrisprGold	Chu et al., 2016a, Graf et al., 2019	N/A
ICE	Synthego, Hsiau et al., 2019	https://www.synthego.com/
ImageJ	NIH imageJ	https://imagej.nih.gov/ij

### Lead Contact and Materials Availability

Further information and requests for resources and reagents should be directed to and will be fulfilled by the Lead Contact, Van Trung Chu (Vantrung.chu@mdc-berlin.de). All unique and/or stable reagents generated in this study are available from the Lead Contact with a completed Materials Transfer Agreement.

### Experimental Model and Subject Details

#### Mice

Cas9 transgenic mice (R26-Cas9iGFP) were reported previously (Chu et al., 2016b). C57BL/6N, and Rag2^−/−^cγ^−/−^ mice were bred in house. Rag2^−/−^ mice were purchased from Taconic Biosciences (NY, USA). All mice were kept in specific pathogen-free facilities. R26-Cas9iGFP, C57BL/6N and Rag2^−/−^ mice used were male or female and 8-12 weeks old. Rag2^−/−^cγ^−/−^ mice used were male and 12-16 weeks old. All animal experiments were approved by the Institution Animal Care and Use Committee (Berlin LaGeSo).

#### Cell lines

HEK293T cells were maintained in DMEM^+/+^ (GIBCO) supplied with 10% FCS (Biochrom) and 25mM HEPES (GIBCO).

#### Primary cells

Cells were isolated from the bone marrow of male R26-Cas9iGFP, C57BL/6 or Rag2^−/−^ mice. Sca1^+^ cells were isolated using the Sca1 enrichment kit according to the manufacturer’s protocol (Milteny Biotec). 2x10^5^ Sca1^+^ cells were cultured in 1ml of serum-free StemSpan™ SFEM II medium (Stemcell technologies) supplied with mouse SCF (50ng/ml), mouse TPO (50ng/ml), mouse Flt3L (50ng/ml) and human IL-11 (50ng/ml) (Peprotech).

### Method Details

#### sgRNA design with CrisprGold

Using CrisprGold, we designed a sgRNA targeting the sequence nearby the Stop codon of mouse *Lmnb1* locus and 2 sgRNAs targeting the pTK-Neo-pA cassette in the Neo-disrupted *Rag2* allele. All of sgRNAs were high specificity with the lowest off-target risk score ≥ 6.

#### rAAV-DJ repair template cloning and rAAV-DJ production

To generate pAAV-Lmnb1-T2A-mCherry and pAAV-Actb-T2A-BFP vectors, the left and right homology arms were amplified from genomic DNA and cloned into the XhoI/EcoRI and AsiSI/KpnI sites of the pTV-T2A-mCherry/BFP vectors, respectively. The NotI-flanked Lmnb1-T2A-mCherry or Actb-T2A-BFP fragment were cloned into the pAAV-DJ vector (Cell Biolabs). To generate the repair templates for the neomycin-disrupted *Rag2* allele, the NotI-flanked wild-type fragment of the Rag2 sequence was amplified from genomic DNA by PCR and cloned into the pAAV-DJ vector.

To produce rAAV-DJ viruses, HEK293T cells were co-transfected with pAAV-DJ, pAAV-DJ-Rep/Cap and pAAV-Helper plasmids using the PEI transfection protocol. 12h later, the medium was replaced with DMEM^+/+^ supplemented with 10% FCS, 25mM HEPES (GIBCO) and 10 μg/ml Gentamycin (Lonza). Three days later, the cell pellet was collected and lysed by 3 cycles of thaw-freeze on dry-ice/ethanol bath (10min per cycle). Then cell lysate was cleared by spinning at 3500rpm for 15min. The cleared supernatant was transferred into new falcon tubes and treated with DNA endonuclease Benzonase (Millipore) for 1h at 37°C. The cell lysate was spun down and the supernatant was loaded into Iodixanol gradient tubes (Beckman) and fractionated by ultra-centrifugation at 58000 rpm for 130min at 18°C, using the type 70Ti rotor (Beckman). The 40% iodixanol layer was collected using an 18 gauge needle and syringe. The supernatant was filtered through a 0.2 μm PES filter and dialyzed overnight twice with PBS at 4°C in dialysis cassettes (Thermo Scientific). Finally, the rAAV supernatant was concentrated by ultra-centrifugal unit (Millipore). The titer of rAAV-DJ viruses was measured by real-time PCR using TaqMan probes specific for the AAV ITR sequence (Life technologies).

#### dsDNA and ssDNA donor template preparation

To generate the dsDNA donor template, the Lmnb1-T2A-mCherry fragment was amplified and cloned into the XhoI/NotI sites of the pTV plasmid. The pTV-Lmnb1-T2A-mCherry vector was digested with XhoI and NotI, and the Lmnb1-T2A-mCherry fragment was purified using the gel extraction kit (Macherey-Nagel). To generate the ssDNA donor template, a nicking endonuclease with a different orientation (Nb.BsrDI) was added to the 5′ end of the Lmnb1-T2A-mCherry fragment by PCR amplification. Then the Nb.BsrDI-containing Lmnb1-T2A-mCherry was cloned into the XhoI/NotI sites of the pTV vector. Depending on the orientation of the Nb.BsrDI endonuclease, the ssDNA-antisense or sense was generated by digestion and gel purification. Briefly, the nicking pTV-Lmnb1-T2A-mCherry was digested using the NotI and Nb.BsrDI enzymes (NEB), and the digested plasmid was precipitated with ethanol. The digested plasmid pellet was resuspended at 2 μg/ul in water. Two volumes of RNA loading buffer (Thermo Scientific) were added to the digested plasmid. The plasmid was denatured at 70°C for 15min, chilled on ice for 1min, loaded into 1.2% agarose gel and run at 120V for 1h. The expected ssDNA band was excised and purified using the QIAGEN gel extraction kit (QIAGEN). The ssDNA donor was finally concentrated using the isopropanol method.

#### sgRNA and RNP electroporation and AAV infection

To generate the sgRNA complexes, crRNA (100pmol, 1.2 μg) and tracrRNA (100pmol, 2.2 μg) were mixed at a 1:1 ratio, incubated at 95°C for 5min and ramped down to room temperature. To generate the RNP complexes, Cas9 proteins (50pmol, 8.2 μg) were mixed with sgRNAs (100pmol, 3.3 μg) at a 1:2 molarity ratio and incubated at 25°C for 10min. The Sca1^+^ cells were cultured for 2 days and then washed 1 time with PBS. 3x10^5^ Sca1^+^ cells were suspended into 20 μL of electroporation buffer containing the synthetic modified sgLmnb1, sgActb (100 (3.3 μg), 150 (4.95 μg) or 200 (6.6 μg) pmol) or RNPs (50pmol (8.2 μg) Cas9 and 100pmol (3.3 μg) sgRNA). After electroporation using the ‘mouse B cell program’ (Lonza), the cells were transferred to pre-warmed SFEM-II medium supplied with cytokines and placed into an incubator at 37°C and 5% CO_2_. 30 min later, the rAAV-DJ particles were added to electroporated cells. The medium was changed on the next day. The HSPCs were analyzed by flow cytometry or harvested for genomic DNA extraction 48h-72h post rAAV-DJ infection for further analysis.

#### LSK cell primary and secondary transplantation

48h after AAV infection, the CRISPR/Cas9-targeted Sca1^+^ cells were collected and washed 3 times with PBS (room temperature). The cells were then resuspended in FACS buffer (PBS/1%BSA) and stained for 15min with Lin, Sca1 and Ckit antibodies. Lin^−^Sca1^+^Ckit^+^ LSK cells were then sorted using Aria sorters (BD Diva). 5x10^4^ sorted LSK cells were mixed with 2x10^6^ Rag2^−/−^c_γ_^−/−^ derived BM carrier cells and intravenously injected into sub-lethally irradiated (5 Gy) Rag2^−/−^c_γ_^−/−^ mice. 2, 4, 8 or 16 weeks after reconstitution, the immune cells in the transplanted mice were analyzed by flow cytometry. For secondary transplantation, 8 weeks post primary reconstitution the donor LSK cells were sorted using Aria sorters (BD Diva) from primary recipient animals, 5x10^4^ sorted donor LSK cells were mixed with 2x10^6^ Rag2^−/−^c_γ_^−/−^ derived BM carrier cells and intravenously injected into sub-lethally irradiated (5 Gy) Rag2^−/−^c_γ_^−/−^ mice. 5 weeks post secondary reconstitution, the immune cells in the transplanted mice were analyzed by flow cytometry.

#### FACS analysis and sorting

For FACS analysis, single cell suspensions were prepared from bone marrow and spleen of the recipient animals and the Fc receptors were blocked by incubation with FcγR antibodies (BioLegend) for 10min. The surface antigens were then stained with fluorescent-conjugated antibodies for 15min. The cells were finally washed with FACS buffer (PBS/1%BSA) and analyzed by BD Fortessa. Cell survival was defined as fraction of live cells (measured by DAPI stain exclusion). The data were analyzed using FlowJo®.

#### Proliferation assay

The Sca1^+^ cells were isolated from R26-Cas9iGFP mice using the Sca1 enrichment kit, 1x10^6^ cells were then washed twice with PBS in RT and labeled with 5 μM CellTrace violet (Invitrogen) at 37°C for 15 min. The labeled cells were washed with serum-free StemSpan™ SFEM II medium (Stemcell technologies). 2x10^5^ cells were cultured with 1ml StemSpan™ SFEM II medium supplied with cytokines and 10 μg/ml Gentamycin (Lonza). Proliferation rates were measured by flow cytometry at day 2 and 4 after CellTrace labeling. The cell division was anlyzed using FlowJo®.

#### Colony forming assay

Two days post targeting the reporter^+^ LSK cells were sorted by FACS and the coloning forming assays were performed using methylcellulose medium M3434 (Stemcell technologies), according to the manufacturer’s protocol. Briefly, 500 sorted LSK cells were resuspended in 1.5ml of methylcellulose-based medium, and then placed into one well of 6 well plate. Cells were incubated 10 days in the incubator at 37°C, 5%CO_2_. Single colonies were manually picked for PCR analysis.

#### PCR, T7EI assay and Sequencing

Genomic DNA from the targeted LSK cells was extracted using the QuickExtract DNA extraction kit (Epicenter) following the manufacturer’s protocol. The targeted sequences were amplified from gDNA by PCR (25-30 cycles) using the Herculase II Fusion DNA Polymerase (Agilent Technology) or LongAmp® Taq 2x master mix (NEB) with gene-specific primers (Table S1). PCR products were separated on 1.2% agarose gels. The expected band was excised and purified by NucleoSpin® Gel and PCR clean-up kit (Macherey-Nagel). For the T7EI assay, 200ng cleaned PCR products were digested with T7EI (NEB) according to the manufacturer’s instructions. Cleaved DNA fragments were separated on 1.5% agarose gels and the DNA concentration of each band was quantified using the ImageJ software (NIH imageJ). Percentage of HR and NHEJ rates was calculated as previously described (Cong et al., 2013). The DNA fragments, amplified by Herculase II and LongAmp® Taq were cloned into Zero blunt plasmids (Invitrogen) and TOPO TA plasmids (Invitrogen). Plasmids were isolated using the NucleoSpin® Plasmid (Macherey-Nagel) and sequenced using Sanger sequencing.

#### Off-target analysis

5 highest risk off-target sites for sgLmnb1 and sgActb were selected using CrisprGold. The on-target and off-target sites were amplified using LongAmp® Taq 2x master mix (NEB) with gene specific primers (Table S1). PCR products were cleaned using AMPure XP beads (Beckman Coulter). 200ng PCR products were digested with T7EI as described above. Cleaved DNA fragments were separated on 2% agarose gels. Frequency of NHEJ was quantified using the ImageJ software (NIH imageJ). For tracking of indels by ICE sequencing analysis, the cleaned PCR products were sequenced using Sanger sequencing and then analyzed by ICE sequencing tool (Synthego). Percentage of indels was calculated based on decomposition agothirm and represented as ICE-score (Hsiau et al., 2019).

### Quantification and Statistical Analysis

p values were computated with Prism 7.0 (GraphPad) using the non-parametric Mann-Whitney test. ^∗^ p < 0.05, ^∗∗^ p < 0.01. For each experiment, 2 mice per genotype were used per biological replicate. Data are shown as mean ± SD values from three independent experiments. The number of biological replicates for each type of experiment is outlined in the figure legends or shown as data points in figures.

### Data And Code Availability

This study did not generate/analyze [datasets/code].

## Supplemental Information

Document S1. Figures S1–S11 and Table S1

Document S2. Article plus Supplemental Information
