# Supplementary material for: Efficient CRISPR/Cas9-Mediated Gene Knockin in Mouse Hematopoietic Stem and Progenitor Cells
Source: Cell Rep. 2019 Sep 24;28(13):3510–3522.e5. doi: 10.1016/j.celrep.2019.08.065 (PMC6899516; doi:10.1016/j.celrep.2019.08.065)
Supplement: Document S1. Figures S1–S11 and Table S1 [file mmc1.pdf]

Cell Reports, Volume *28*

Supplemental Information

Efficient CRISPR/Cas9-Mediated Gene Knockin

in Mouse Hematopoietic Stem and Progenitor Cells

Ngoc Tung Tran, Thomas Sommermann, Robin Graf, Janine Trombke, Jenniffer Pempe, Kerstin Petsch, Ralf Kühn, Klaus Rajewsky, and Van Trung Chu

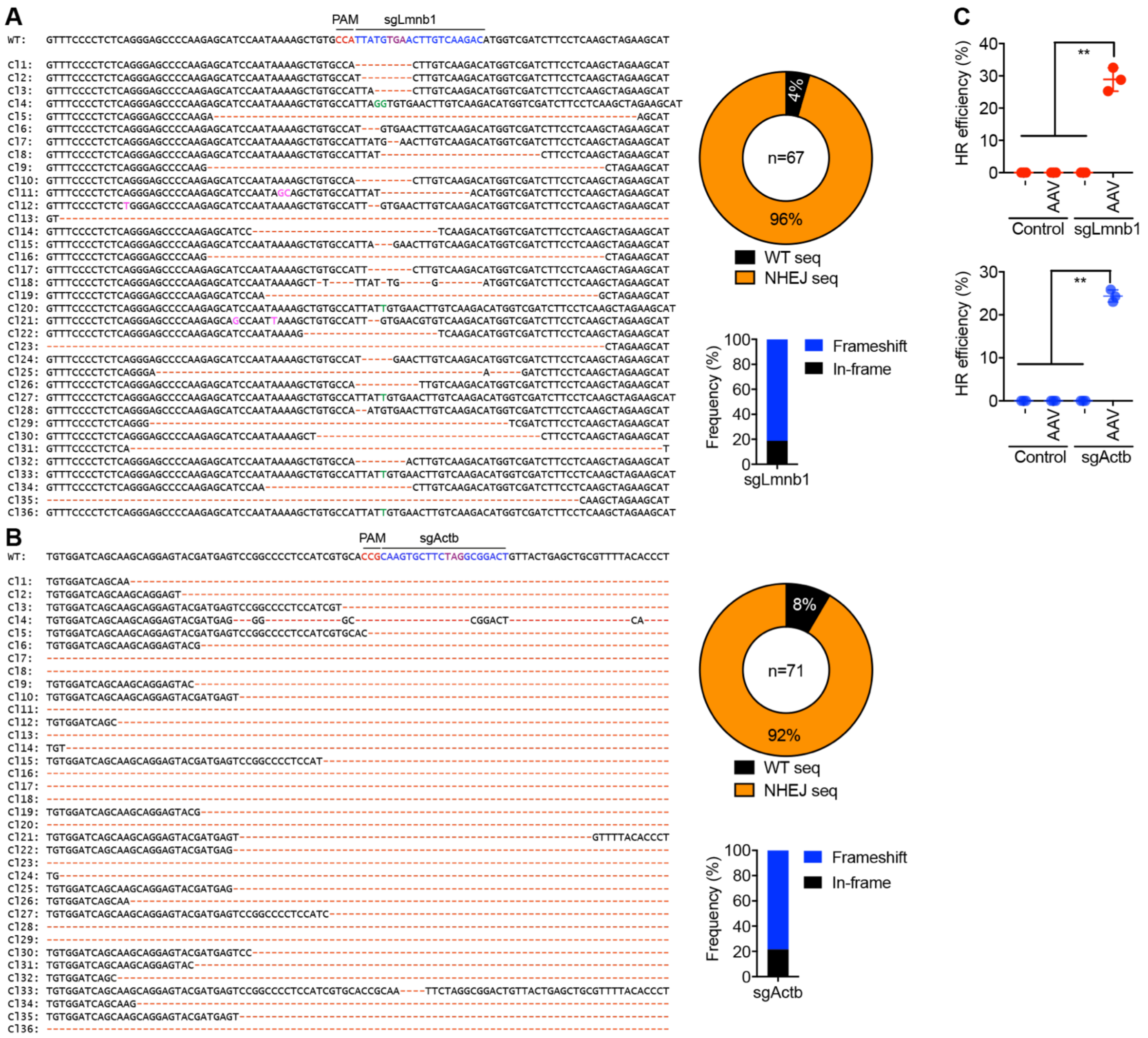

Figure S1. Indel analysis of the targeted *Lmnb1* and *Actb* loci in mouse HSPCs, Related to Figure 1. Activated Cas9-HSPCs were electroporated with sgLmnb1 (**A**) or sgActb (**B**). 3 days post gene editing, the LSK cells were sorted and the targeted sequences were amplified by PCR, cloned and sequenced. PAM signal, sgRNA and Stop codon are indicated in red, blue and magenta, respectively. The pie charts and histograms show the efficiencies of NHEJ events and type of NHEJ mutations. (**C**) Summary of HR efficiency in the targeted loci for the data shown in main Figure 1D. Quantification of HR frequency represent means ± SD (**p<0.01, Mann-Whitney test). Data are representative for at least 3 independent experiments.

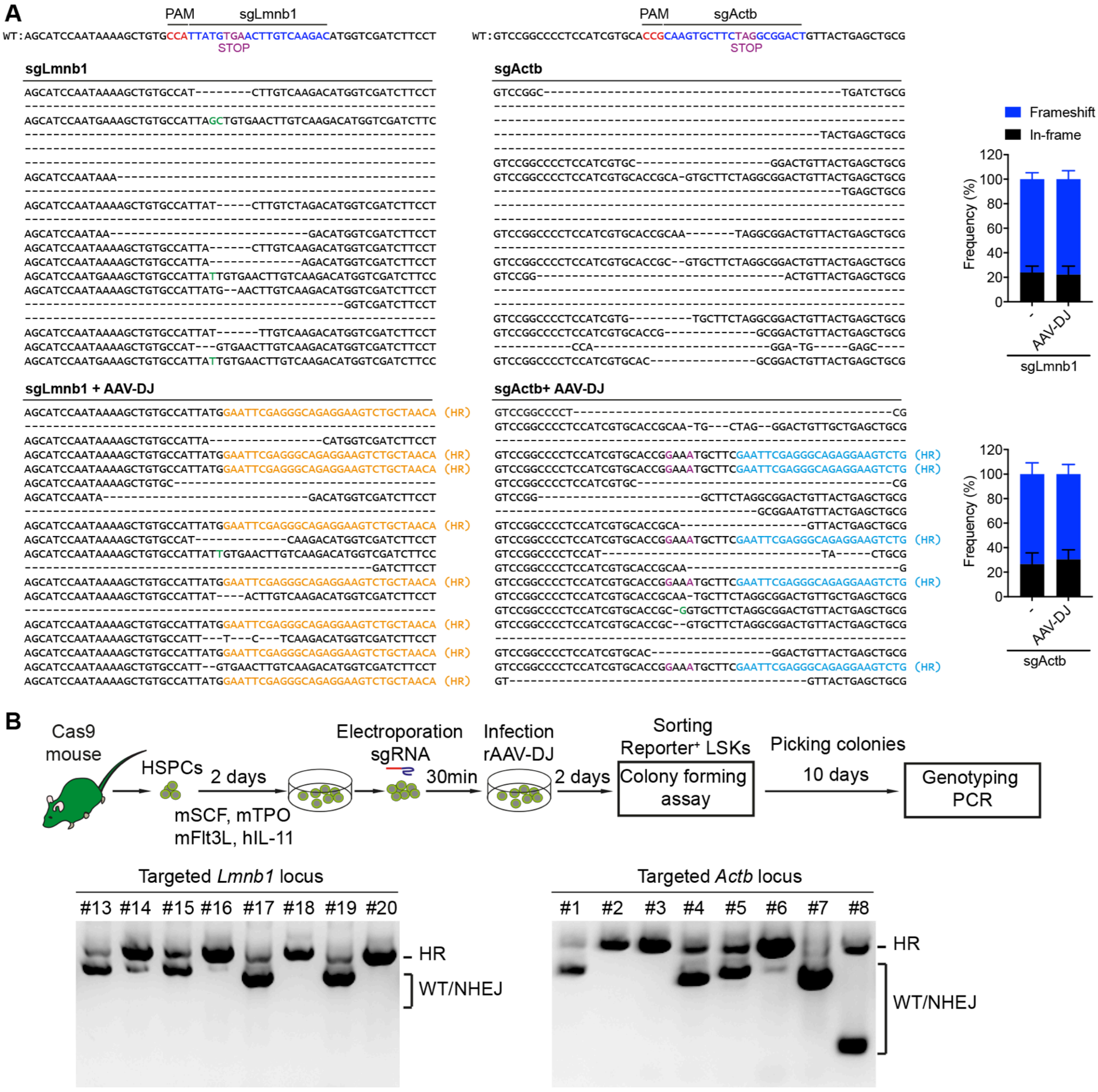

Figure S2. Quantification of HR and NHEJ frequencies in the targeted HSPCs, Related to Figure 1. (**A**) Sequences amplified from the *Lmnb1* (left) and *Actb* (right) loci in the targeted HSPCs that were treated with sgRNAs only (top) or sgRNA and AAV-DJ donor vectors (bottom). The sequences in orange and blue were corrected by HR. Frequencies of frame-shift (blue) and in-frame (black) mutations in NHEJ events were quantified (right panel, bar graphs). (**B**) Experimental scheme to determine mono- and bi-allelic HR events using PCR genotyping for individual reporter^{+} colonies. Two days post targeting, the reporter^{+} LSK cells were sorted and colony forming assays were performed. Ten days later, single colonies were picked for genotyping PCR. The targeted *Lmnb1* (left) and *Actb* (right) loci were amplified using the primer sets indicated in main Figure 1A.

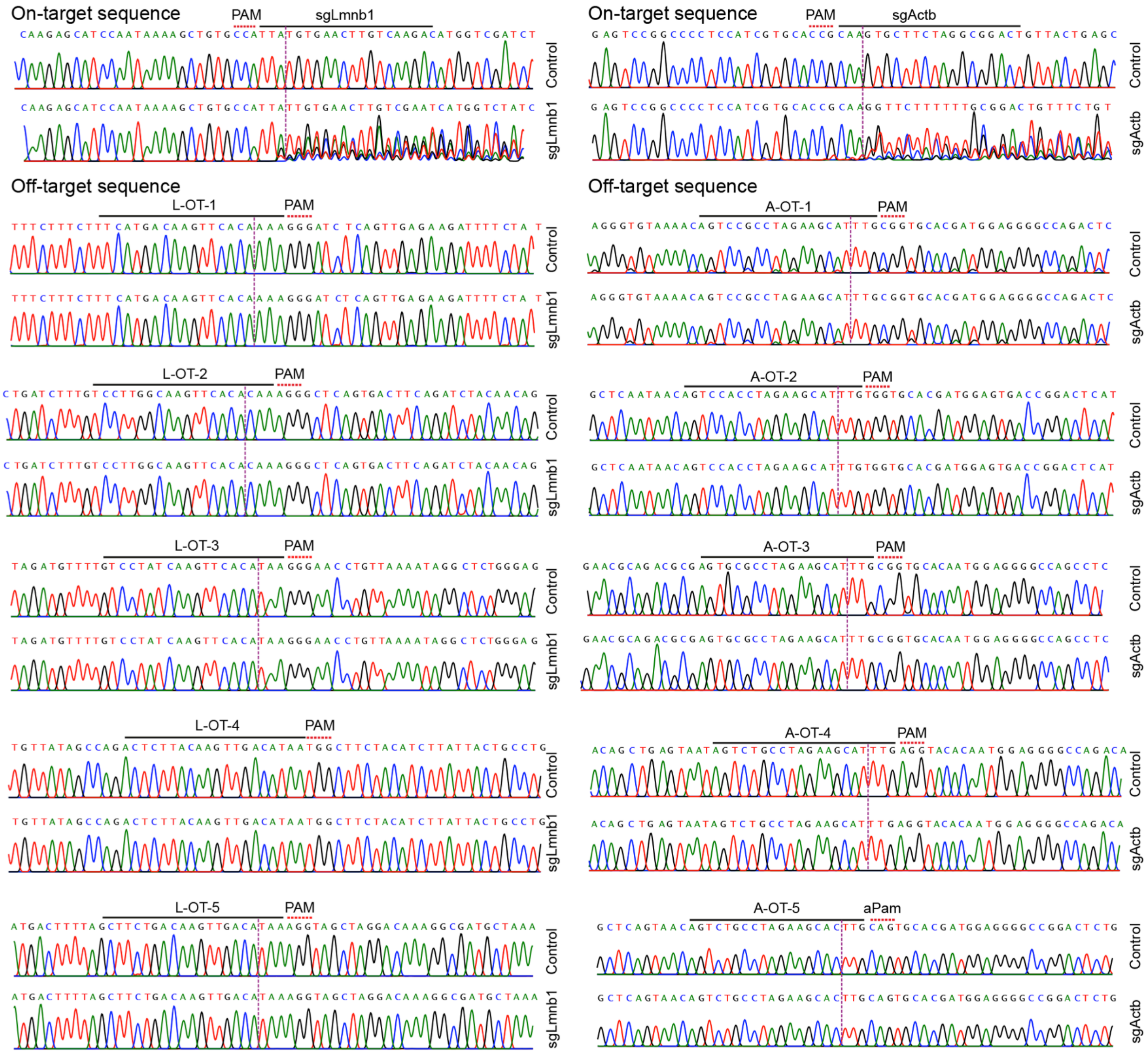

Figure S3. Sequencing of on- and off-target sites of sgLmnb1 and sgActb, Related to Figure 2. The on- and (top 5) off-target sites of sgLmnb1 (left) and sgActb (right) were amplified from untreated HSPCs (control) or HSPCs that were treated with the indicated sgRNAs and sequenced 3 days post editing. The dashed red lines highlight the PAMs or alternative PAMs (aPam), the adjacent black lines indicate the on/off-target, and the dashed magenta lines indicate the predicted potential cut sites.

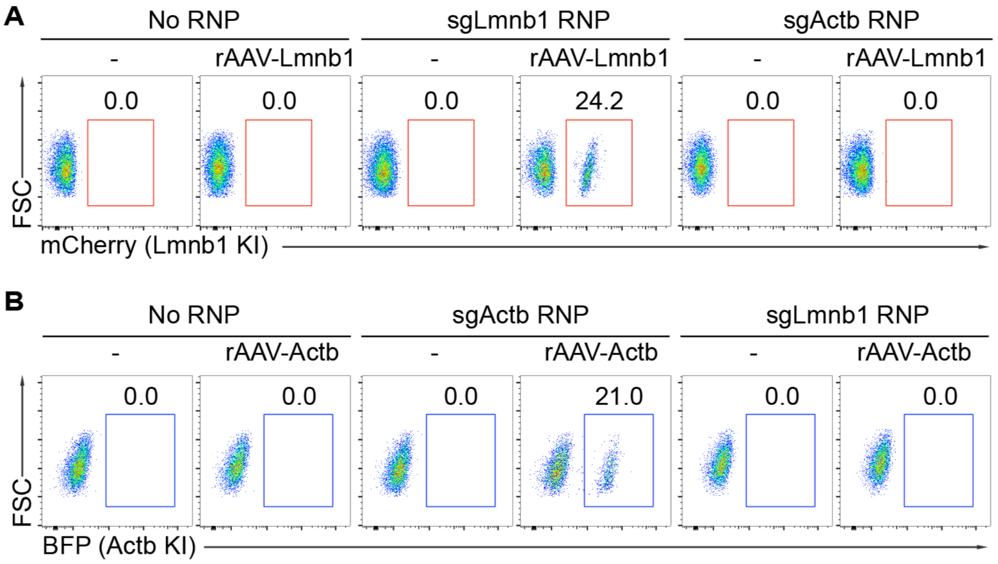

Figure S4. HR-mediated gene insertion in wild-type HSPCs, Related to Figure 2. Activated-Sca1^{+} HSPCs from C57BL/6 mice were electroporated with RNPs containing sgLmnb1 (**A**) or sgActb (**B**) and subsequently infected with the indicated AAV-DJ donor vectors. The frequencies of mCherry^{+} (**A**) and BFP^{+} (**B**) LSK cells were analysed by flow cytometry 3 days post targeting.

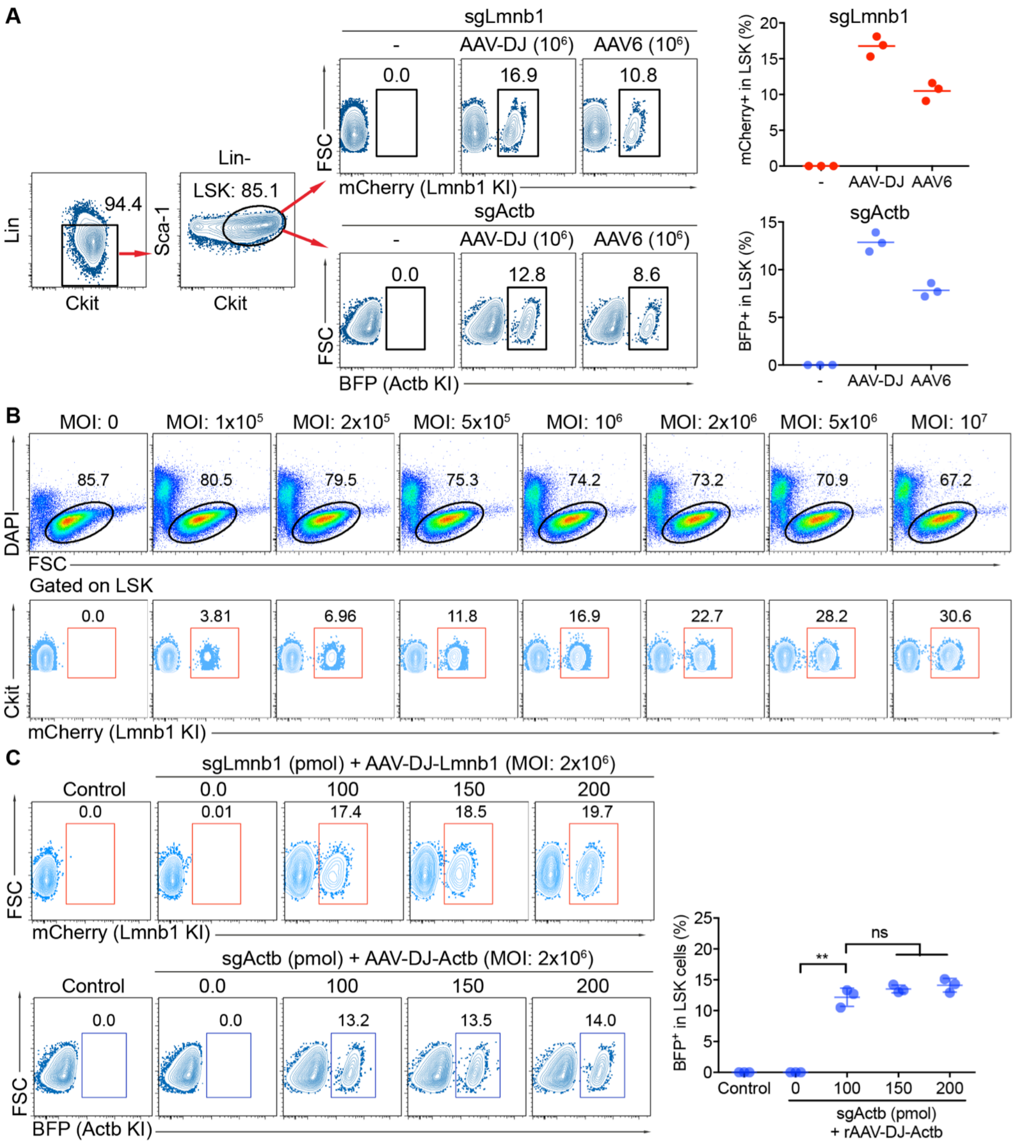

Figure S5. Comparison of AAV-DJ and AAV6 and titration of AAV-DJ vectors and sgRNA, Related to Figure 3. (**A**) Activated Cas9-HSPCs were electroporated with the indicated sgRNAs and infected with AAV-DJ or AAV6 donor vectors at a MOI of $1x10^6$ GC/cell. Using the gating strategy, the frequencies of $mCherry^+$ and BFP^+ cells in the targeted Cas9-HSPCs were quantified. Data are based on 3 independent experiments. (**B**) Activated Cas9-HSPCs were electroporated with sgLmnb1 and infected with the indicated doses of rAAV-DJ-Lmnb1 donor virus. The cell viability (as measured by $DAPI^-$) and the HR efficiency in LSKs were determined by flow cytometry. (**C**) Activated Cas9-HSPCs were electroporated with the indicated doses of sgLmnb1 (upper panel) or sgActb (lower panel) before infection with rAAV-DJ-Lmnb1 or rAAV-DJ-Actb donor viruses (MOI of $2x10^6$ GC/cell). The frequencies of $mCherry^+$ and BFP^+ cells were determined by flow cytometry. The histogram summarizes the frequencies of BFP^+ cells in the Actb-targeted HSPCs from 3 independent experiments. Data show means ± SD (** p<0.01, Mann-Whitney test); ns: not significant.

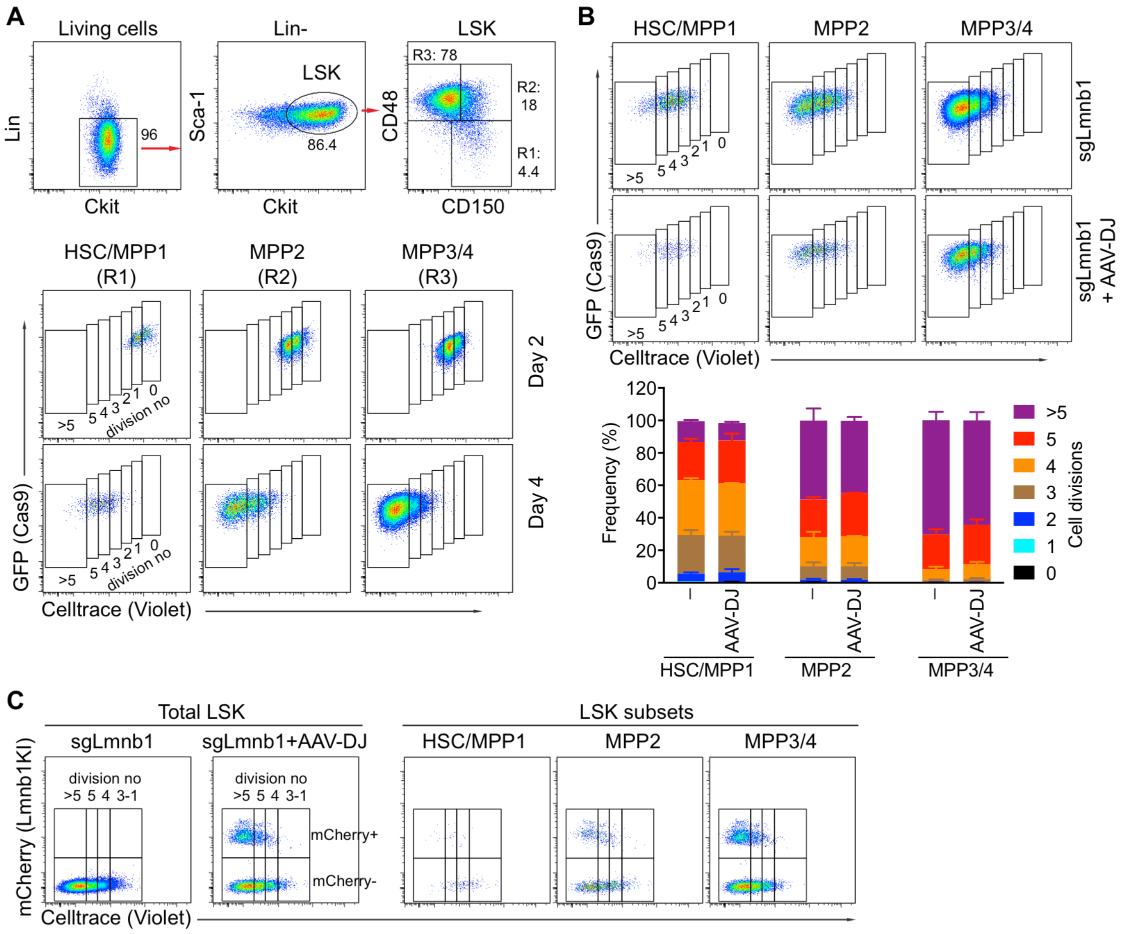

Figure S6. HR efficiencies and proliferation rates in mouse LSK subsets, Related to Figure 3. (**A**) Sca1^{+} Cas9-HSPCs were labeled with CellTrace violet and activated. Proliferation rates were determined by flow cytometry on day 2 and 4 post labeling. Gating on the HSC/MPP1 (R1: CD48^{-}CD150^{+}), MPP2 (R2: CD48^{+}CD150^{+}) and MPP3/4 (R3: CD48^{+}CD150^{-}) LSK subsets, cell division was determined by analysing the dilution of CellTrace violet. (**B**) 2 days after stimulation, the labeled cells were electroporated with sgLmnb1 and infected with the AAV-DJ-Lmnb1 donor vectors. FACS analysis shows numbers of cell division in each LSK subset. Graph summarizes the fractions of cells in each LSK subset that underwent the indicated number of cell division. (**C**) FACS analysis shows frequencies of gene knock-in (mCherry^{+}) versus cell division in the total LSK cells (left) and each LSK subset (right).

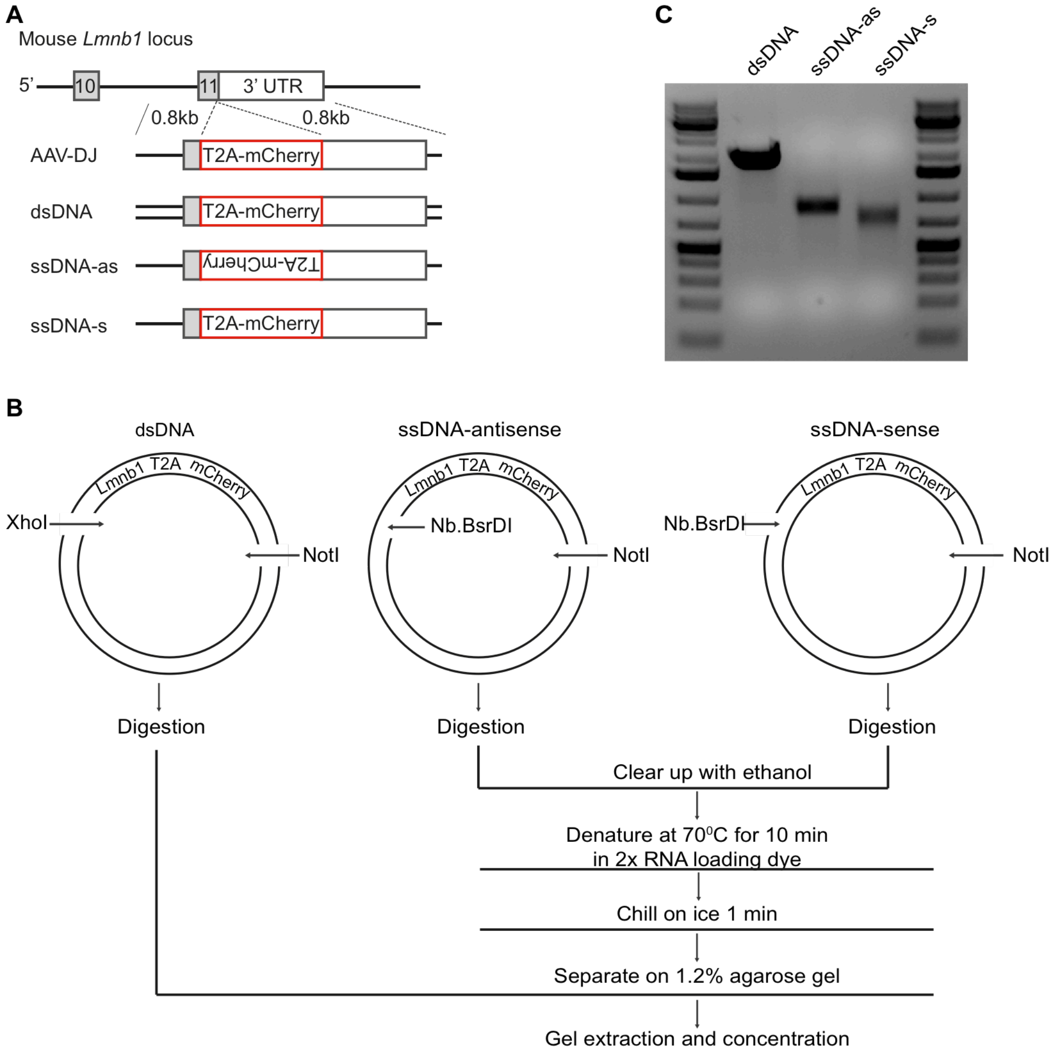

Figure S7. Generation of non-viral dsDNA and ssDNA donor templates, Related to Figure 4. (**A**) Scheme of gene insertion into the *Lmnb1* locus using the indicated donor template types with 0.8 kb homology arms. (**B**) To generate Lmnb1-mCherry dsDNA donor templates, the XhoI/NotI-flanked Lmnb1-T2A-mCherry fragment was digested with XhoI and NotI restriction enzymes. This fragment was then separated on an agarose gel and purified by gel extraction. To generate the Lmnb1-mCherry ssDNA (antisense and sense), a nicking nuclease (Nb. BsrDI) recognition site was added into the 5' end of Lmnb1-T2A-mCherry fragment. Depending on the orientation of the nicking nuclease recognition site, the antisense or sense ssDNA is produced. The nicking plasmids were digested with NotI and nicking Nb. BsrDI restriction enzymes. The digested plasmids were then purified by ethanol precipitation and resuspended in denaturing buffer. The digested plasmids were denatured at 70°C for 10 min and chilled on ice for 1 min. The ssDNA HR templates were separated on agarose gel, and the expected band was excised and purified by gel extraction kit. (**C**) Gel of the obtained dsDNA and ssDNA donor templates.

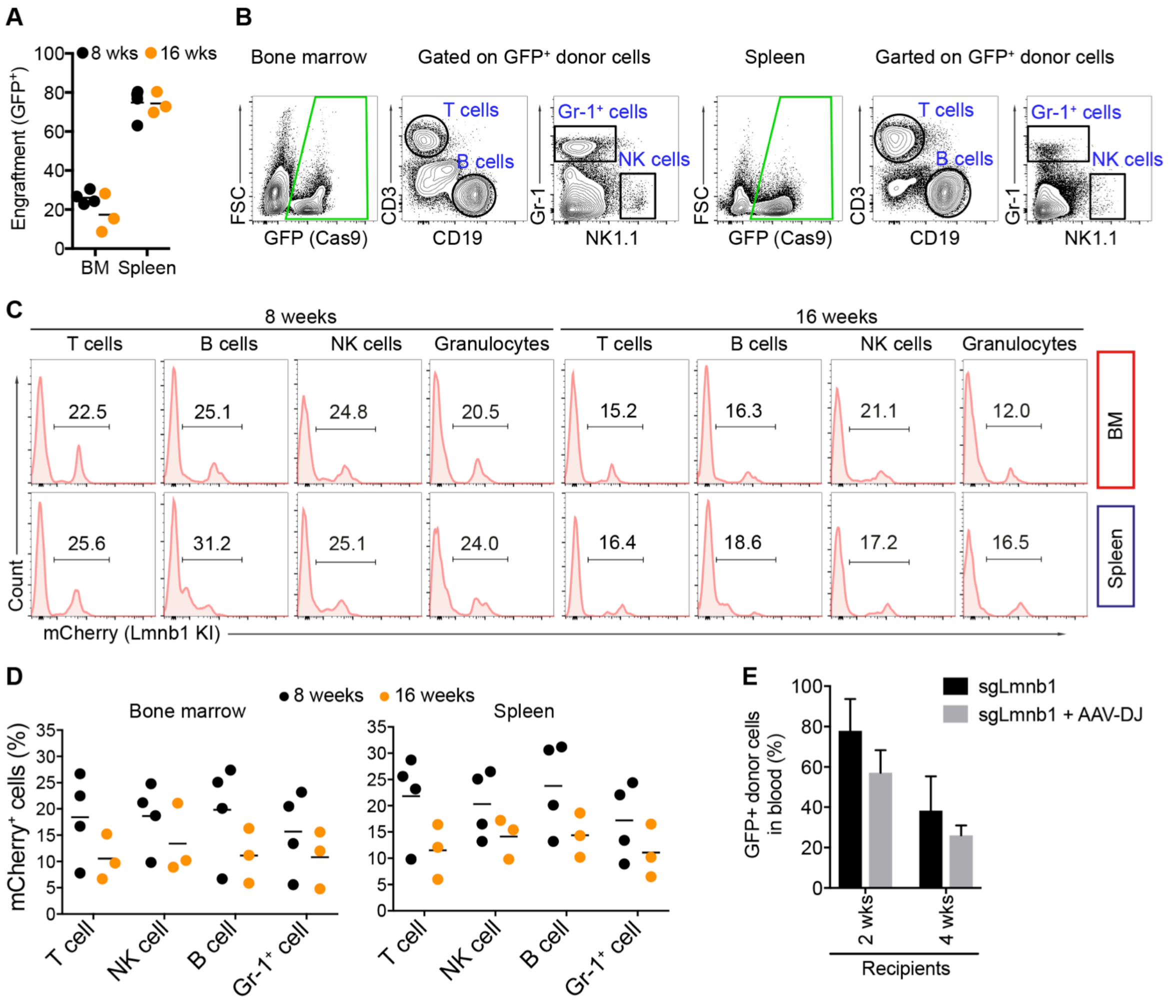

Figure S8. The targeted HSPCs contribute to the immune system, Related to Figure 5. (**A**) Frequencies of GFP$^+$ donor cells in the bone marrow and spleen of the recipients after 8 (black, n=4) and 16 weeks (orange, n=3) of reconstitution. (**B**) Gating strategy to analyse the T, B, NK, and Gr-1$^+$ cells among GFP$^+$ donor cells in the bone marrow and spleen of the recipient animals 8 weeks post transplantation. (**C**) Histograms showing the percentages of mCherry$^+$ cells among T cells, B cells, NK cells and Granulocytes (Gr-1$^+$) in the bone marrow (upper row) and spleen (lower row) of the recipient animals after 8 and 16 weeks of reconstitution. (**D**) Summary of percentages of mCherry$^+$ cells among the indicated immune cell lineages in the bone marrow (left) and spleen (right) of the recipient animals after 8 weeks (black, n=4) and 16 weeks (orange, n=3) post reconstitution. (**E**) Graph summarizes frequencies of GFP$^+$ donor cells in the blood of the recipient mice (n=4) that reconstituted with either sgLmnb1-treated (black) or sgLmnb1/AAV-DJ-treated LSK cells (grey), at 2 and 4 weeks post reconstitution.

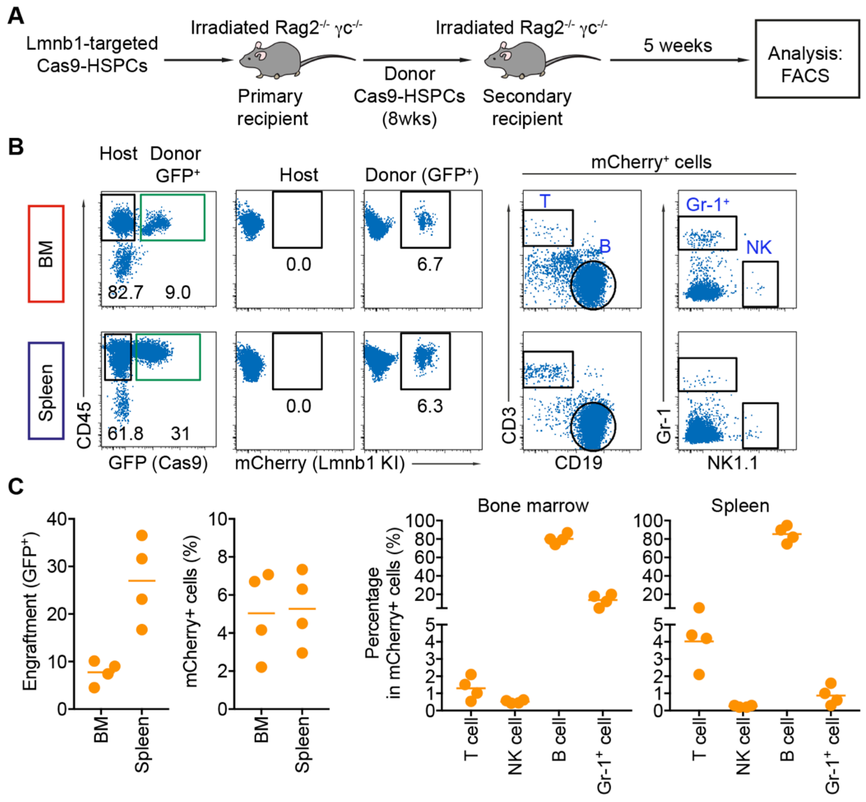

Figure S9. Secondary reconstitution of the Lmnb1-targeted HSPCs, Related to Figure 5. (A) Experimental scheme of secondary transplantation of the Lmnb1-targeted HSPCs into irradiated Rag2$^{-/-}$c$_{\gamma}^{-/-}$ mice. The targeted Cas9-HSPCs (GFP^{+}) were sorted from the recipient animals 8 weeks post primary reconstitution. These cells were then transplanted into secondary recipients (n=4). Immune cell lineages were analysed by flow cytometry 5 weeks post secondary reconstitution. (**B**) FACS analysis shows the percentage of GFP^{+} donor and knocked-in mCherry^{+} cells in the bone marrow and spleen of the secondary recipients. Percentages of T, B, NK, and Gr-1^{+} cells among mCherry^{+} cells were analyzed by gating on mCherry^{+} cells. (**C**) Summary of data based on 4 recipient mice 5 weeks post secondary reconstitution.

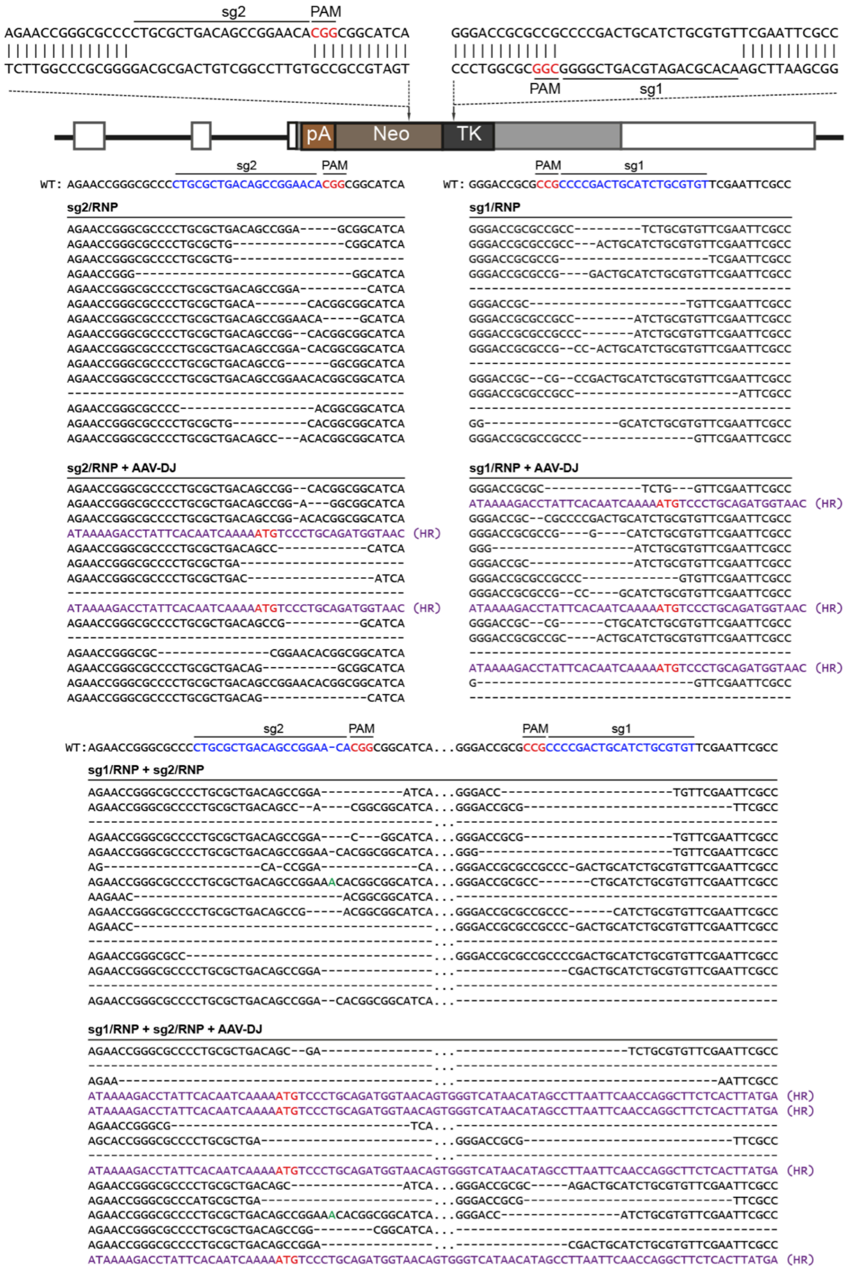

Figure S10. HR and NHEJ frequencies in Rag2-repaired HSPCs, Related to Figure 6. Scheme of a Neo-disrupted *Rag2* allele including the TK promoter, neomycin and the polyA sequence. The sgRNA target sequences of sg-1 and sg-2 are highlighted in blue and the respective PAM sequences in red. Lower panel: sequencing data of the amplified targeted loci showing correct HR (HR, magenta) and indel patterns in the cells that received RNPs only (top) or RNPs and AAV-DJ donor vectors (bottom).

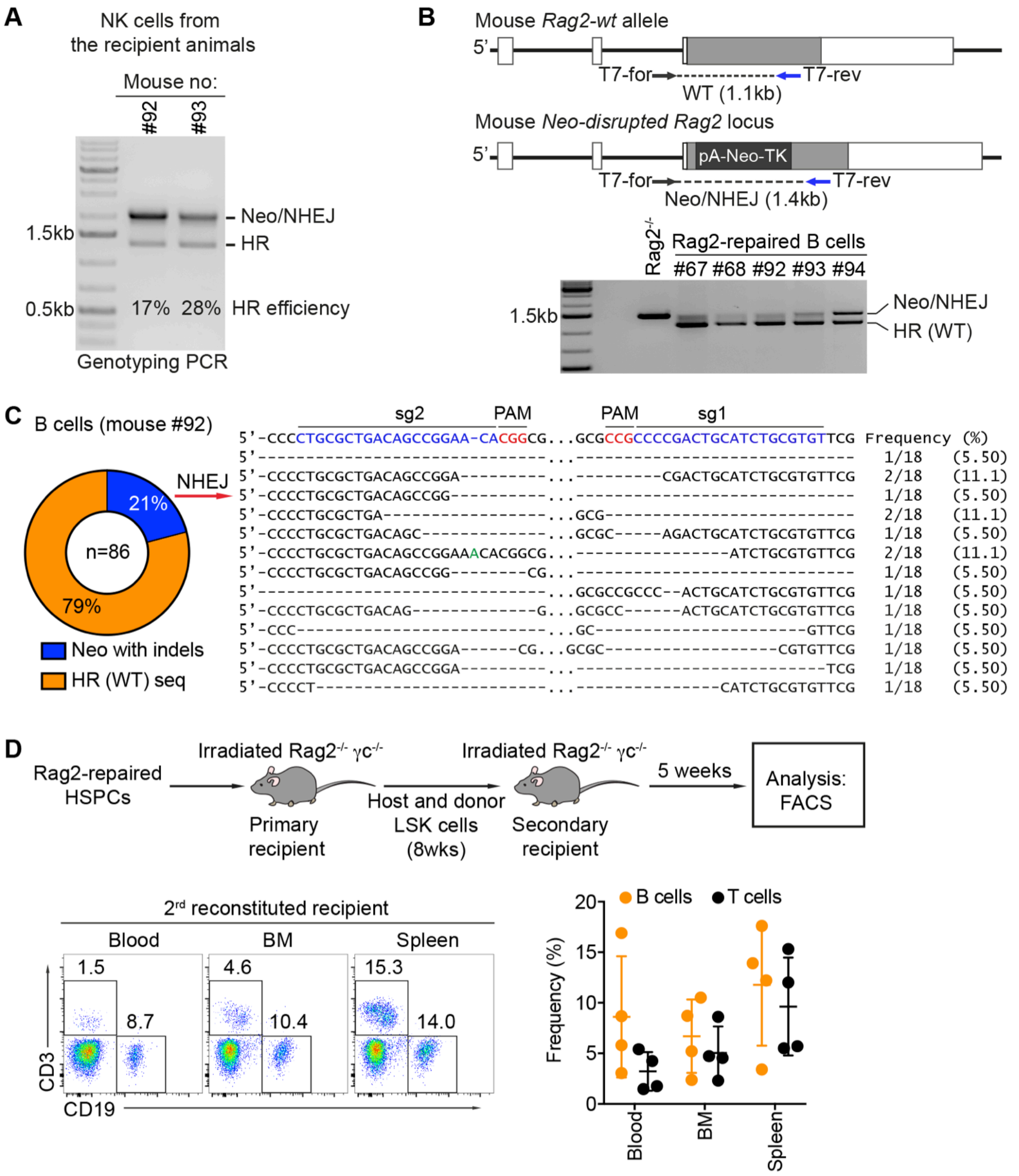

Figure S11. Molecular analysis of Rag2-repaired NK and B cells, and secondary reconstitution of Rag2-repaired HSPCs, Related to Figure 7. (**A**) HR frequencies in NK cells from 2 recipient animals (#92 and #93) 8 weeks post reconstitution. gDNA was amplified by PCR using the primer sets as indicated in main Figure 6A. The numbers reflect the HR efficiency based on band quantification. (**B**) HR frequencies in B cells from 5 recipient animals (#67, 68, 92, 93 and 94) 8 weeks post reconstitution. The targeted locus was amplified using the PCR primers as indicated in the scheme. As a control, gDNA from a $Rag2^{-/-}$ mouse was used. (**C**) Indel diversity analysis in B cells. The frequencies of HR and NHEJ events were quantified by sequencing (pie chart). In the cases of NHEJ, the diversity of indels was analysed. (**D**) Experimental scheme of secondary reconstitution of Rag2-repaired HSPCs. The LSK cells, including host and donor HSPCs, were sorted from the recipient animals 8 weeks post primary reconstitution and transplanted into secondary recipients. 5 weeks post secondary reconstitution, B and T cells were analysed in the blood, bone marrow and spleen of the recipients. Summary of the data from blood, bone marrow (BM) and spleen of 4 recipient animals 5 weeks post secondary reconstitution.

Table S1. Sequences of primers used in this study, related to Star Methods.

PCR Primers	Sequence (5'-3')	Related to Figures
Lmnb1-T7-For	AATCTTAACTGTTTACAGGCCTAGGTCAGCT	**Figure S1, Figure 2 and Figure S3**
Lmnb1-T7-Rev	CAGTACAGTTAGCTCAGTGTCAATAATTCACATCT	
Actb-T7-For	CTGTGGTTGTCAGAGCAACCTTCTAGGTT	**Figure S1, Figure 2 and Figure S3**
Actb-T7-Rev	CAACCAACTGCTGTCGCCTTCACCGTTCCA	
Lmnb1-5HA-For	ACTCTTCCAGTGTGGGTCAGCCAAGCTTCT	**Figure 1 and Figure S2**
Lmnb1-T7-Rev	CAGTACAGTTAGCTCAGTGTCAATAATTCACATCT	
Actb-5HA-For	TCTCAGATCTATCCATACAGTTTCACCTGC	**Figure 1 and Figure S2**
Actb-T7-Rev	CAACCAACTGCTGTCGCCTTCACCGTTCCA	
L-OT-1-For	TGCCTCTACTTGCCGAGCCATCTCACCAAT	**Figure 2 and Figure S3**
L-OT-1-Rev	CCAGGACCACCAGACCAGGGATGGCACTGT	
L-OT-2-For	GGCTGCTACATACGTGCCTGTGAGTGCCTC	**Figure 2 and Figure S3**
L-OT-2-Rev	CCTTCCTAAGTGGTTTCCTTCAGCTCTGCT	
L-OT-3-For	AGGACTGCCTTGTGCCAGATCTGTAAGAGA	**Figure 2 and Figure S3**
L-OT-3-Rev	GTATTGGACTCCTAGAAGCAGGTGGGACAA	
L-OT-4-For	CACTGGCACTAGCTGTTTCATCTTCCTGCC	**Figure 2 and Figure S3**
L-OT-4-Rev	GCTCTCAGCTATGGCTCCAGCATCAAGCCT	
L-OT-5-For	GAGAAAGTGCTTTACAGCTGGATCTCACGA	**Figure 2 and Figure S3**
L-OT-5-Rev	ATCAATGGTCAACACCGGGTACAATTGCGT	
A-OT-1-For	GGATGTTTGCTTCAACCGACTGCCTTCGCC	**Figure 2 and Figure S3**
A-OT-1-Rev	CATGTACCCAGGCATTGCTGACAGGATGCA	
A-OT-2-For	CCAGTTTTTAAATCTCGAGTCCAAAGCAC	**Figure 2 and Figure S3**
A-OT-2-Rev	CAGGCATTGCTGACCTGATGCAGGAGATCA	
A-OT-3-For	ACAGGCGCCGGCTGCAGTGGCGGCGATGGA	**Figure 2 and Figure S3**
A-OT-3-Rev	GGAACACGCAGGCACGTGACACTCTTGTCT	
A-OT-4-For	AAGAAGCCATGCCAGGGTTATCTCAGGGTT	**Figure 2 and Figure S3**
A-OT-4-Rev	CCTAGTTGATGAGCACAGTGTGGGTGACCT	
A-OT-5-For	TGCGCAAGTTAGGTTTTGTCAAAGAAAGGGT	**Figure 2 and Figure S3**
A-OT-5-Rev	CCAGAATGAATTTGGATGAGGACAGAGATGA	
Rag2-T7-For	CCACTAAAATAGGTCCAAGCTGCTGCCACA	**Figure 6 and Figure S11**
Rag2-T7-Rev	GGAGTCTCCATCTCACTGATTTCAATCGTG	
Rag2-5HA-For	ACTACACTTCCGTTACTATGTGCACCAGCG	**Figure 6, Figure 7, Figure S10 and Figure S11**
Rag2-WT-Rev	ACTATACACCACGTCAATGGAATGGCCGTA	
Rag2-Neo/NHEJ-Rev	ACAAGGGCAGTGTGGTTTTCAAGAGGAAGC	
